# Recovery of hippocampal functions and modulation of muscarinic response by electroacupuncture in young diabetic rats

**DOI:** 10.1038/s41598-017-08556-z

**Published:** 2017-08-22

**Authors:** Marzia Soligo, Sonia Piccinin, Virginia Protto, Francesca Gelfo, Maria Egle De Stefano, Fulvio Florenzano, Erica Berretta, Laura Petrosini, Robert Nisticò, Luigi Manni

**Affiliations:** 1 Institute of Translational Pharmacology, Consiglio Nazionale Delle Ricerche (CNR), Rome, Italy; 2 European Brain Research Institute (EBRI), Rita Levi-Montalcini Foundation, Rome, Italy; 3 I.R.C.C.S., Santa Lucia Foundation, Rome, Italy; 4 Department of Systemic Medicine, University of Rome “Tor Vergata”, Rome, Italy; 5 Department of Biology and Biotechnology “Charles Darwin”, Sapienza University of Rome, Laboratory affiliated to Istituto Pasteur-Fondazione Cenci Bolognetti, Rome, Italy; 6 Department of Psychology, Faculty of Medicine and Psychology, University “Sapienza” of Rome, Rome, Italy; 7 Department of Biology, University of Rome Tor Vergata, Rome, Italy

## Abstract

The muscarinic receptor response to acetylcholine regulates the hippocampal-related learning, memory, neural plasticity and the production and processing of the pro-nerve growth factor (proNGF) by hippocampal cells. The development and progression of diabetes generate a mild cognitive impairment reducing the functions of the septo-hippocampal cholinergic circuitry, depressing neural plasticity and inducing proNGF accumulation in the brain. Here we demonstrate, in a rat model of early type-1 diabetes, that a physical therapy, the electroacupuncture, counteracts the diabetes-induced deleterious effects on hippocampal physiology by ameliorating hippocampal-related memory functions; recovering the impaired long-term potentiation at the dentate gyrus (DG-LTP) and the lowered expression of the vesicular glutamate transporter 1; normalizing the activity-dependent release of proNGF in diabetic rat hippocampus. Electroacupuncture exerted its therapeutic effects by regulating the expression and activity of M1- and M2-acetylcholine muscarinic receptors subtypes in the dentate gyrus of hippocampus. Our results suggest that a physical therapy based on repetitive sensory stimulation could promote hippocampal neural activity, neuronal metabolism and functions, and conceivably improve the diabetes-induced cognitive impairment. Our data can support the setup of therapeutic protocols based on a better integration between physical therapies and pharmacology for the cure of diabetes-associated neurodegeneration and possibly for Alzheimer’s disease.

## Introduction

Dysfunctions in hippocampus-related behaviour and neural plasticity characterize both Alzheimer’s disease and diabetic encephalopathy^1–3^ that share common hallmarks, such as accumulation of beta-amyloid, of hyper-phosphorylated tau^4, 5^ and of the pro-nerve growth factor (proNGF) in the brain^6, 7^. Basal forebrain cholinergic neurons (BFCN) in the medial septum project to the hippocampus, regulating the activity of its internal excitatory and inhibitory circuitries, as well as those of its external inputs^8^. The BFCNs activity modulates hippocampal plasticity and regulates the production, secretion and processing of proNGF by hippocampal cells^9, 10^ providing a sort of “on demand” supply of the pro-neurotrophin. This in turn regulates remote BFCN metabolism^11^ but also directly influences hippocampal neurons survival^12, 13^, functions and plasticity^14^.

The pro-apoptotic or pro-neurotrophic action of proNGF depends on the balance among different possible receptor complexes^15^ and on the activity of the extracellular proteases cascade responsible for proNGF conversion into the neurotrophic mature NGF (mNGF)^10^. We recently demonstrated a prevalence of proNGF and a lower mNGF/proNGF ratio, in the brain of early diabetic rats^6^. ProNGF is also predominant in the brain of Alzheimer’s patients^7^, indicating that it could reliably participate in the generation of neuronal sufferance and cognitive impairment.

A physical therapy, the electroacupuncture (EA), based on repetitive and controlled stimulation of sensory afferents, regulates brain NGF content in diabetic rats^5^. It is known that acupuncture, as well as physical exercise, which shares with electroacupuncture common neurophysiological substrates^16^, could regulate brain activity, not only in pain-related brain areas^17^ but also in the limbic system^18^. Electroacupuncture positively affects hippocampal neurogenesis^19^ and counteracts hippocampus-related cognitive deficits in diabetic rats^20^. Nevertheless, the mechanism(s) underlying these electroacupuncture effects, on hippocampal physiology, are still largely unexplored.

Type-1 diabetes could early affect cognitive performance in young people, generating a mild cognitive deficit that in turn could progress in diabetic encephalopathy^2, 4, 21^. Our work aimed at studying the impact of early type-1 diabetes and electroacupuncture on activity-regulated hippocampal functions, namely the generation of LTP at dentate gyrus (DG-LTP) and the release and extracellular processing of proNGF. We also explored the mechanistic hypothesis that electroacupuncture corrects the diabetes-induced dysregulation in DG-LTP and proNGF release by acting on the hippocampal muscarinic responsivity.

## Results

### Diabetes-induced hippocampal cell loss and decrease in nuclear size was reversed by electroacupuncture

**Figure 1:**
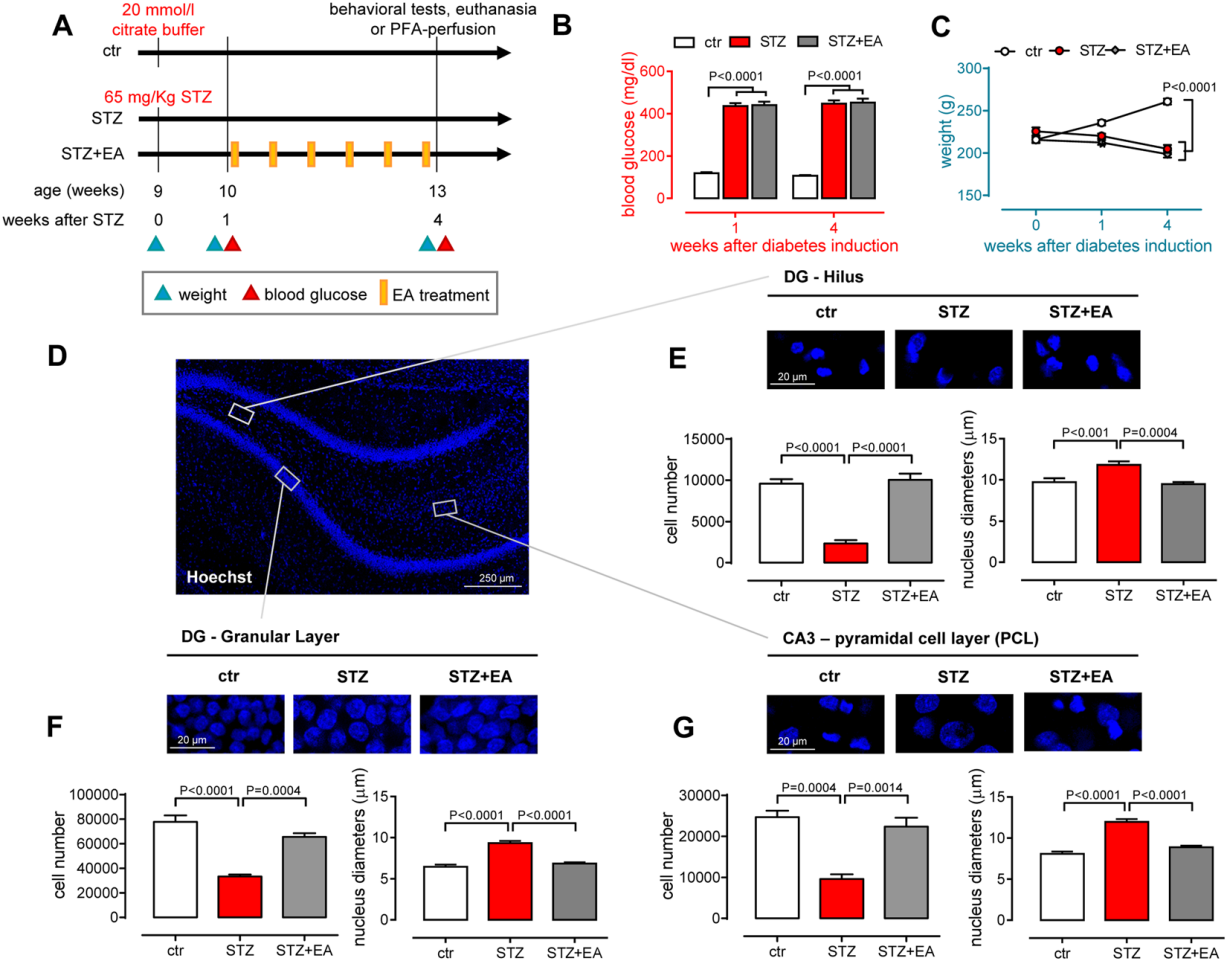
Electroacupuncture partially rescues diabetes-induced hippocampal cell loss. (**A**) Study design. (**B**) Blood glucose and (**C**) body weight measured 1 and 4 weeks after STZ treatment (mean ± SEM; n = 25 for each group). Repeated measures two-way ANOVA followed by Bonferroni multiple comparisons (P values shown in figure). (**D**) Representative picture of the dentate gyrus after Hoechst nuclear staining. Boxes highlight the regions were the analysis, shown in panels E–G, was performed. (**E–G**) Quantitative stereological analysis of the total number of cells and nuclear morphometric analysis in the dentate gyrus (DG; hilus and granular layer) and CA3 pyramidal layer of the rostral septal region of the hippocampus. In the upper part of the panels, representative pictures for each of the experimental groups. In the lower part of the panels, graphs depicting the results of the cell count and nuclear size analysis (means ± SEM of the estimated total cell number, n = 4 animals for each experimental group). One-way ANOVA followed by Bonferroni multiple comparisons (P values shown in figure).

We used a widely accepted animal model of type 1 diabetes^22^. The study design is depicted at Fig. 1A. We checked the establishment of diabetes by analysing blood glucose concentration and body weight over 4 weeks (Fig. 1B,C). Streptozotocin (STZ) increased blood glucose levels (Fig. 1B). These values persisted significantly high also in the STZ+EA group, suggesting that electroacupuncture did not affect the overall glucose metabolism. Rats’ body weight significantly decreased (Fig. 1C) in both STZ and STZ+EA groups compared to controls.

Diabetes induces cell loss in the hippocampus^3^. We explored such feature in three hippocampal areas, to verify whether electroacupuncture was able to counteract the diabetes-induced effects. We evaluated by stereological analysis the total number of cells in the granular layer (GL) and hilus (HL) of the DG and in the CA3-pyramidal cell layer (PCL) (Supplementary Fig. S1A,B), counting Hoechst-labelled nuclei (Fig. 1D–G). STZ overall reduced the number of total cells (Fig. 1E–G), which recovered after electroacupuncture treatment. Reduced number of Hoechst-labelled nuclei was accompanied by alterations in nuclear shape and staining texture, a common feature in all considered regions. The highly compact GL structure was looser in diabetic rats compared to controls, with surviving cells displaying particularly enlarged nuclei (Fig. 1E–G). Mean nuclear size returned to control levels after electroacupuncture treatment (Fig. 1E–G).

To assess whether the observed changes in hippocampal total cell number could reflect similar changes in neurons, we counted the number of NeuN^+^ and NeuN^−^ cells in all three areas (Supplementary Fig. S1A,B). The number of neurons (NeuN^+^) in the GL decreased after streptozotocin and recovered in the STZ+ EA group (Supplementary Fig. S1C,D). The number of NeuN^−^ cells decreased in the GL in both the STZ and STZ+EA groups (Supplementary Fig. S1C,D). In the HL, both NeuN^+^ and NeuN^−^ cell number was lower in the STZ group compared to controls (Supplementary Fig. S1C,E), with only the number of NeuN^−^ cells recovering in the STZ+EA group. In the PCL, NeuN^+^ and NeuN^−^ cell number decreased after streptozotocin (Supplementary Fig. S1F,G) and only the number of NeuN^+^ cells normalized after electroacupuncture. Thus, the hippocampus was overall affected by cell loss, particularly neurons, in the DG and CA3 areas, at an early stage of diabetes development. This could be the result of increased apoptosis, as indirectly indicated by nuclear morphology, and of decreased DG neurogenesis, as indicated by our preliminary evidences on the decrease of doublecortin (DCX)-stained cells in the diabetic DG (Supplementary Fig. S2A,B). The rescuing effects of electroacupuncture were evident on cell morphology (i.e. nucleus size), on the total cell number and also on the number of DCX-expressing cells in the DG (Supplementary Fig. S2A,B).

### Electroacupuncture improved diabetes-induced impairments in memory, LTP and vGlut1 content in the dentate gyrus

**Figure 2:**
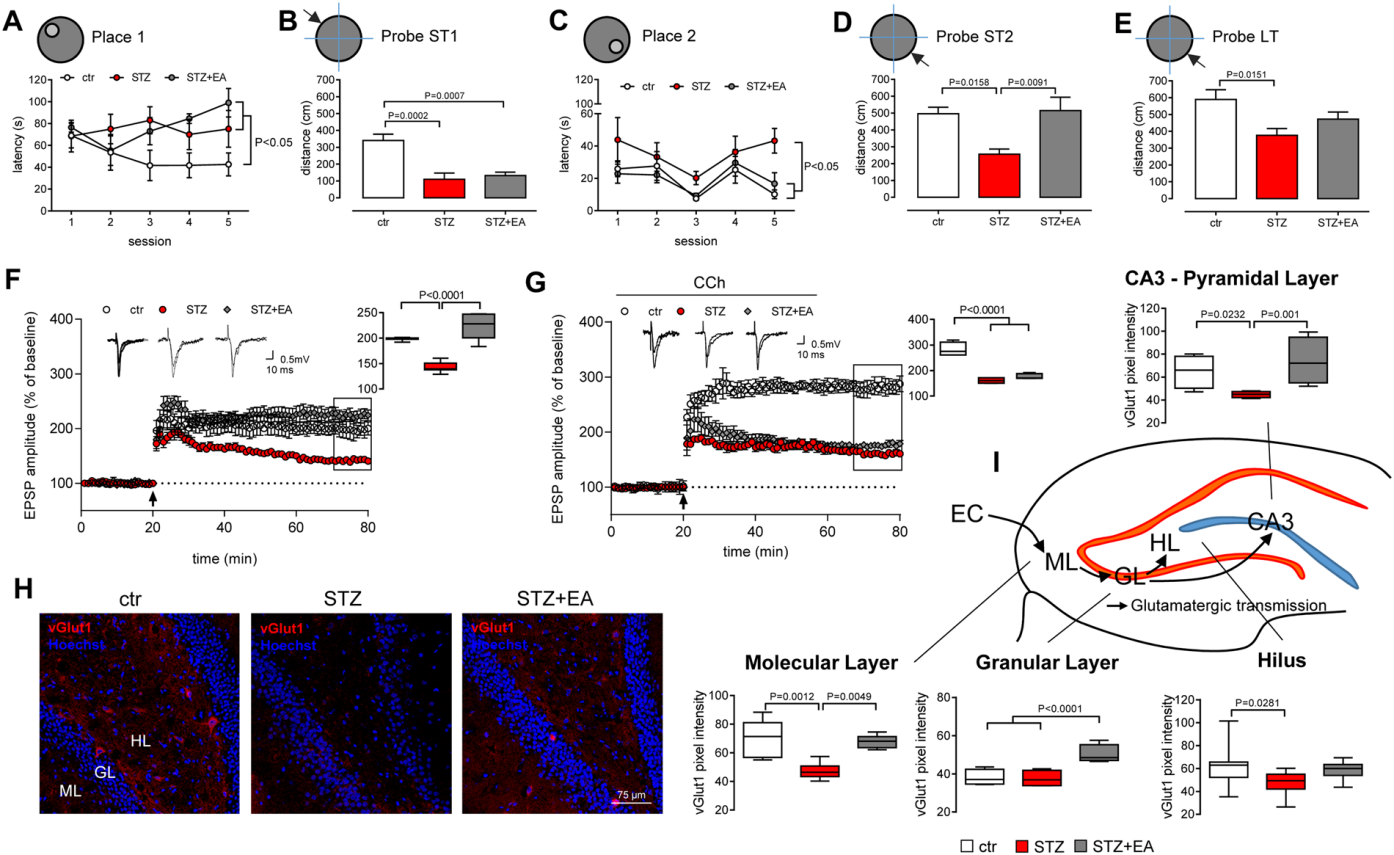
Electroacupuncture improves diabetes-induced impairments in memory, LTP and glutamatergic transmission in the dentate gyrus. (**A–E**) Effects of electroacupuncture (EA) treatment in diabetic animals (STZ) tested in the Morris Water Maze. Latencies to reach the platform in Place 1 (**A**) and Place 2 (**C**) and distances swam in the previously rewarded (platform) quadrant in Short-Term Probe 1 (Probe ST1; **B**), in Short-Term Probe 2 (Probe ST2; **D**), and in Long-Term Probe (Probe LT; **E**) are depicted (means ± SEM; n = 7 for each experimental group). Repeated measures two-way ANOVA followed by Bonferroni multiple comparison test (P values shown in figure). (**F–G**) Long-term potentiation at perforant-pathway (DG-LTP) in absence (**F**) or presence (**G**) of the muscarinic agonist Carbachol (CCh) in the superfusion bath. Field excitatory postsynaptic potentials (fEPSP) were recorded and expressed as the percentage of the pre-tetanus baseline. Changes in fEPSP slopes before and 60 min after the induction of LTP by high-frequency stimulation (HFS) of the medial perforant pathway (black arrow) are shown (means ± SEM; n = 8 for each experimental group). The insets represent typical fEPSP recordings; calibration bars: 0.5 mV, 10 ms. The average of DG-LTP magnitude 50–60 min after HFS is presented on the right side of the panels (median ± interquartile range, whiskers: min. and max.). One-way ANOVA and Bonferroni multiple comparison (P values shown in figure). (**H**) Representative images of vGlut1 immunolocalization in the dentate gyrus of control (ctr), STZ and STZ+EA treated rats. Abbreviations: ML-molecular layer, GL-granular layer, HL-hilus, PCL-pyramidal cell layer. (**I**) Mean pixel intensity of vGlut1 immunofluorescence in the molecular layer, granular cell layer and hilus of the DG and the pyramidal cell layer of the CA3 area (means ± SEM; n = 8 fields, 4 animals for each experimental group). One-way ANOVA followed by Bonferroni multiple comparison test, P values shown in figure. The cartoon illustrates the hippocampal glutamatergic circuitry where immunofluorescence analysis was performed.

Electroacupuncture may improve hippocampus-related cognitive deficits^20^. We investigated, by the Morris water maze (MWM) the early diabetes-induced memory deficits and verified whether electroacupuncture was able to counteract the eventual amnesic impairment. On day 1, in the Place 1, both STZ and STZ+EA groups showed higher latencies to reach the platform in comparison to controls (Fig. 2A; details on statistics at online Supplementary Table 2), indicating that streptozotocin administration induced a significant impairment not reversed by electroacupuncture. Consistently, in the short-term probe 1 phase, STZ and STZ+EA groups swam less than controls in the previously rewarded (platform) quadrant (Probe ST1; Fig. 2B). Otherwise, on day 2, in the Place 2, STZ group showed higher latencies in comparison to both ctr and STZ+EA groups (Fig. 2C; Supplementary Table 2), indicating that the streptozotocin-induced impairment was still present in the second testing day. At this stage, STZ+EA group did not show significant amnesic impairment and exhibited latencies not different from those of the controls (Fig. 2C). In the Probe ST2, diabetic rats swam less in the previously rewarded quadrant in comparison to both ctr and STZ+EA groups (Fig. 2D). Finally, on day 3, in the long-term probe phase, STZ group continued to swim less in the previously rewarded quadrant (Fig. 2E). This finding indicated that the diabetes-induced memory impairment was still present at long term, although ameliorated compared to the previous day, so that the STZ group performance was not different by the one of the STZ+EA group (Fig. 2E). Once more, STZ+EA group showed a long-term memory performance not different by ctr group (Fig. 2E), indicating that electroacupuncture treatment succeeded in counteracting the impairing diabetes effects.

Synaptic plasticity and the hippocampal LTP are major cellular mechanisms underlying learning and memory^23^. We explored the possibility that diabetes may alter the excitatory circuits that drive the hippocampal functions. DG-LTP data and their pairwise comparisons are summarized in Table 1. As reported^24^, the magnitude of DG-LTP was lowered by diabetes (Fig. 2F; Table 1). Notably, electroacupuncture fully restored DG-LTP in diabetic rats (Fig. 2F; Table 1), indicating that electroacupuncture rescuing effects on behavioural functions was associated with a recovery in hippocampal synaptic plasticity. As a first assessment on the role of muscarinic neurotransmission in our model, we evaluated long-term potentiation at dentate gyrus (DG-LTP) in the presence of the muscarinic agonist carbachol (100 nM; Fig. 2G), that is known to facilitate excitatory neurotransmission in the hippocampus^25^. Carbachol enhanced the DG-LTP in control rats (Table 1, row 2 *vs* row 1). Application of carbachol slightly ameliorated the diabetes-induced decrease in DG-LTP (Table 1, row 2 *vs* row 1). Surprisingly, the presence of carbachol, in the superfusion bath, diminished DG-LTP in the STZ+EA group (Table 1, row 2 *vs* row 1), which was not different from the one measured in the STZ group (Fig. 2G; Table 1, row 2). These findings suggest that in diabetic rats the electroacupuncture effects on hippocampal synaptic plasticity relies on muscarinic receptor functions.

**Table 1:**
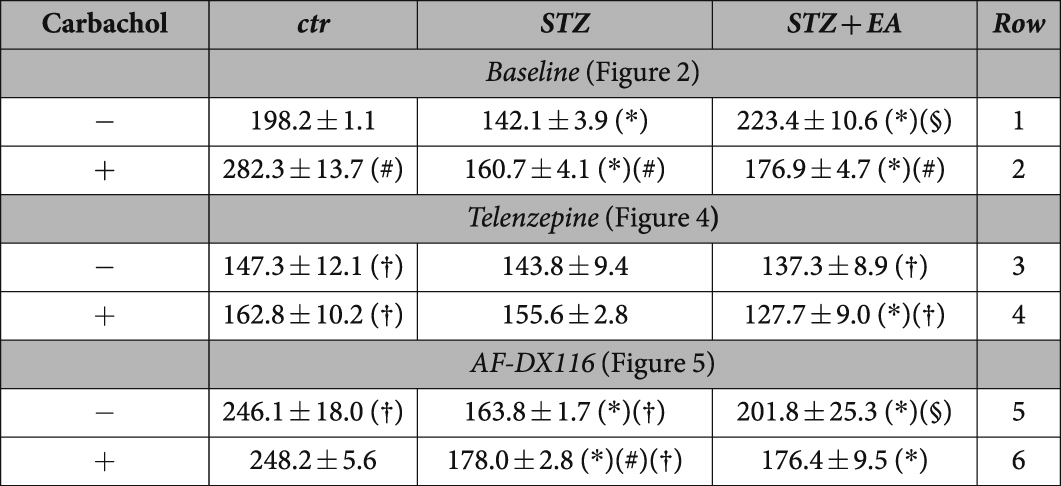
Summary of DG-LTP data presented in Fig. 2, 4, 5 and pairwise comparisons between different groups.

Data, presented as mean ± S.E.M., are percentage increase in fEPSP amplitude, having set the pre-tetanus baseline at 100%. Statistics: *Intra-experiment:* One-way ANOVA followed by Bonferroni multiple comparison test. *P < 0.05 vs controls; §P < 0.05 vs STZ (comparisons within the same row). *Inter-experiment 1:* Unpaired t-test. #P < 0.05 +carbachol vs −carbachol in the same experimental group (comparisons: row 2 vs row 1; row 4 vs row 3; row 6 vs row 5). *Inter-experiment 2:* Unpaired t-test. †P < 0.05 +antagonist vs -antagonist (baseline) in the same experimental group (comparisons: row 3 vs row 1; row 5 vs row 1; row 4 vs row 2; row 6 vs row 2). n = 8 indicates the number rats. One hippocampal slice per animal was used for recordings.

We also studied whether functional changes in the DG excitatory transmission were associated with alterations in tissue distribution of a major marker of glutamatergic transmission, the vesicular glutamate transporter-1 (vGlut1; Fig. 2H,I). Hippocampal content and distribution of vGlut1 was affected by both diabetes and electroacupuncture (Fig. 2H,I). vGlut1 immunolabeling in the ML and in the HL of STZ and STZ+EA groups (Fig. 2H) appeared as a diffuse staining, when compared to controls, that display a specific vGlut1 staining clustered at cell bodies. Protein expression levels were estimated by the mean pixel intensity of the vGlut1 immunofluorescence. After streptozotocin, vGlut1 mean pixel intensity did not vary in the GL, but decreased in all of the other hippocampal regions (Fig. 2I). After electroacupuncture, values either returned to control levels (ML, HL and PCL) or became higher than controls (GL: Fig. 2I). Overall, the confocal microscopy analysis on vGlut1 suggested a decrease in the glutamate transport in diabetic rats that partially recovered after EA treatment.

### proNGF expression was modified by diabetes and electroacupuncture, and modulated by muscarinic receptors activity

**Figure 3:**
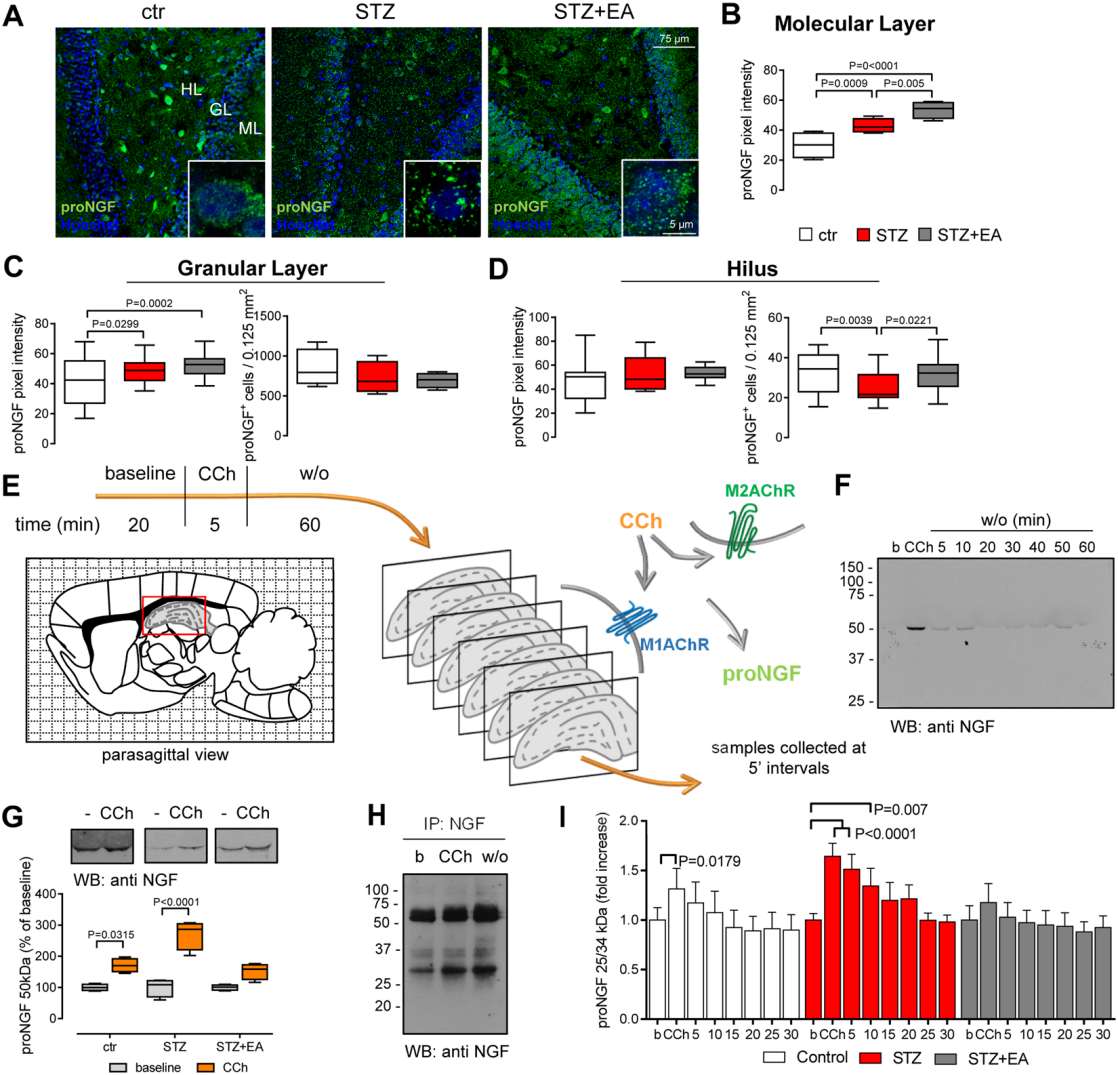
Diabetes and electroacupuncture treatment differentially modulate proNGF expression in the hippocampus. (**A**) Representative images of proNGF immunolocalization in the dentate gyrus (DG) of the hippocampus. Insets: Higher magnifications showing proNGF immunolocalization within DG granule cells. (**B–D**) Mean pixel intensity and the number of proNGF immunopositive (proNGF^+^) cells in different areas of the DG (means ± SEM; n = 8 fields, 4 animals for each experimental group). One-way ANOVA followed by Bonferroni multiple comparison, P values shown in figure. (**E**) Cartoon depicting the experimental procedures performed in superfusion experiments and the possible carbachol (CCh) action on proNGF release by hippocampal cells. (**F**) Representative Western blot (WB) illustrating the time-course of carbachol-stimulated release of the 50 kDa proNGF from hippocampal slices of streptozotocin-treated rats. (**G**) Carbachol-stimulated proNGF release from hippocampal slices. Densitometry analysis of the WB (cropped representatives shown in the upper side of the panel; full-length blots are shown in online Supplementary material) illustrates the differences in carbachol-stimulated proNGF release among the different experimental groups. Data are expressed as percentage of the respective baselines (median ± interquartile range, whiskers: min. and max.). Two-way ANOVA followed by Bonferroni multiple comparisons, P values shown in figure. (**H**) WB of hippocampus superfusates after NGF-immunoprecipitation, revealing the presence of a 25 and a faint 34 kDa proNGF bands. (**I**) CCh-stimulated 25/34 kDa proNGF release from hippocampal slices measured by proNGF ELISA. Data (means ± SEM; n = 6 for each experimental group), represent the percentage of the respective baseline. Repeated-measure ANOVA followed by Bonferroni multiple comparison; P values shown in figure. Abbreviations: ML-molecular layer, GL-granular layer, HL-hilus, b-baseline, w/o-washout.

As the following, we studied proNGF distribution in the DG of control and treated rats. Figure 3A displays representative images of hippocampal slices immunolabeled for proNGF. The overall immunofluorescence intensity increased throughout the entire section in both STZ and STZ+EA groups, as shown by the heavy punctate immunolabeling sparse among the cells. Differently, intracellular immunolabeling appeared to decrease, especially in the HL, after diabetes induction, followed by a slight recovery after electroacupuncture. Insets in each picture detail the distribution of the cellular immunolabeling in the GL: in the controls, proNGF immunofluorescence occurred in small puncta, mainly distributed within the cell bodies. After streptozotocin, larger and more widespread clumps of immunoreactivity were evident, suggesting an increase in both the amount and size of proNGF secretory granules. Electroacupuncture after streptozotocin reduced the size of proNGF-positive (proNGF^+^) granules, indicating a switch toward control conditions. The pixel intensity of the proNGF immunolabeling was increased by diabetes in the ML (Fig. 3B) and GL (Fig. 3C), while it was not affected in the HL (Fig. 3D). Electroacupuncture further increased the mean pixel intensity in the ML (Fig. 3B) and GL (Fig. 3C). Conversely, the number of proNGF^+^ cells decreased after streptozotocin only in the HL (Fig. 3D), returning to control levels after electroacupuncture (Fig. 3D). These data indicate a possible diabetes-induced re-distribution of proNGF toward secretory compartments in both cell soma and dendritic terminals in the ML.

The production, release and extra-cellular processing of proNGF, in the hippocampus, is regulated by the BFCNs activity^10^. Thus, we turned out to a pharmacological stimulation of *ex-vivo* superfused hippocampus slices, to link the cholinergic input with the proNGF release and potential processing (Fig. 3E). A brief exposure of hippocampal slices to carbachol induced a transient increase in a 50 kDa proNGF isoform in the superfusion medium (Fig. 3F). When the hippocampus was exposed to carbachol, a little increase in the 50 kDa proNGF release was found in the control group, while a significant three-fold increase was observed in the STZ group (Fig. 3G). NGF-immunoprecipitation (Fig. 3H) revealed the presence, in the superfusion media, of a 34 kDa (faint) and a 25 kDa bands, most probably representing the proNGF-A and proNGF-B transcripts respectively^26^. Carbachol transiently increased the 25/34 kDa proNGF in the superfusates (Fig. 3I), in the controls. Carbachol stimulated a more robust and sustained proNGF release in the STZ group, that was absent in the STZ+EA group (Fig. 3I). Thus, the muscarinic challenge induced proNGF release from hippocampal slices that was modulated by electroacupuncture.

We also explored whether diabetes and electroacupuncture influenced the protease machinery responsible for the maturation and degradation of proNGF^10^. The carbachol-induced plasminogen and tissue plasminogen activator (tPA) release, from hippocampal slices, were increased in healthy animals (Supplementary Fig. S3), while no significant variations were found in both the STZ and STZ+EA groups. The activity of the matrix metalloproteinase (MMP)-2 and MMP-9 in the superfusates, as revealed by gelatine zymography (Supplementary Fig. S3), was increased in the STZ group and was normalized in the STZ+EA group. Thus, diabetes affected the extracellular protease cascade mainly by increasing MMPs activity, while electroacupuncture counteracted the MMPs over-activity and modulated the muscarinic-regulated release of tPA, a factor upstream to MMPs in the protease activation cascade^10^. These data are consistent with an increased mNGF degradation (by MMPs) in diabetes that could shift toward a correct proNGF maturation and mNGF activity after electroacupuncture.

### M1AChR modulated hippocampal functions after diabetes and electroacupuncture

**Figure 4:**
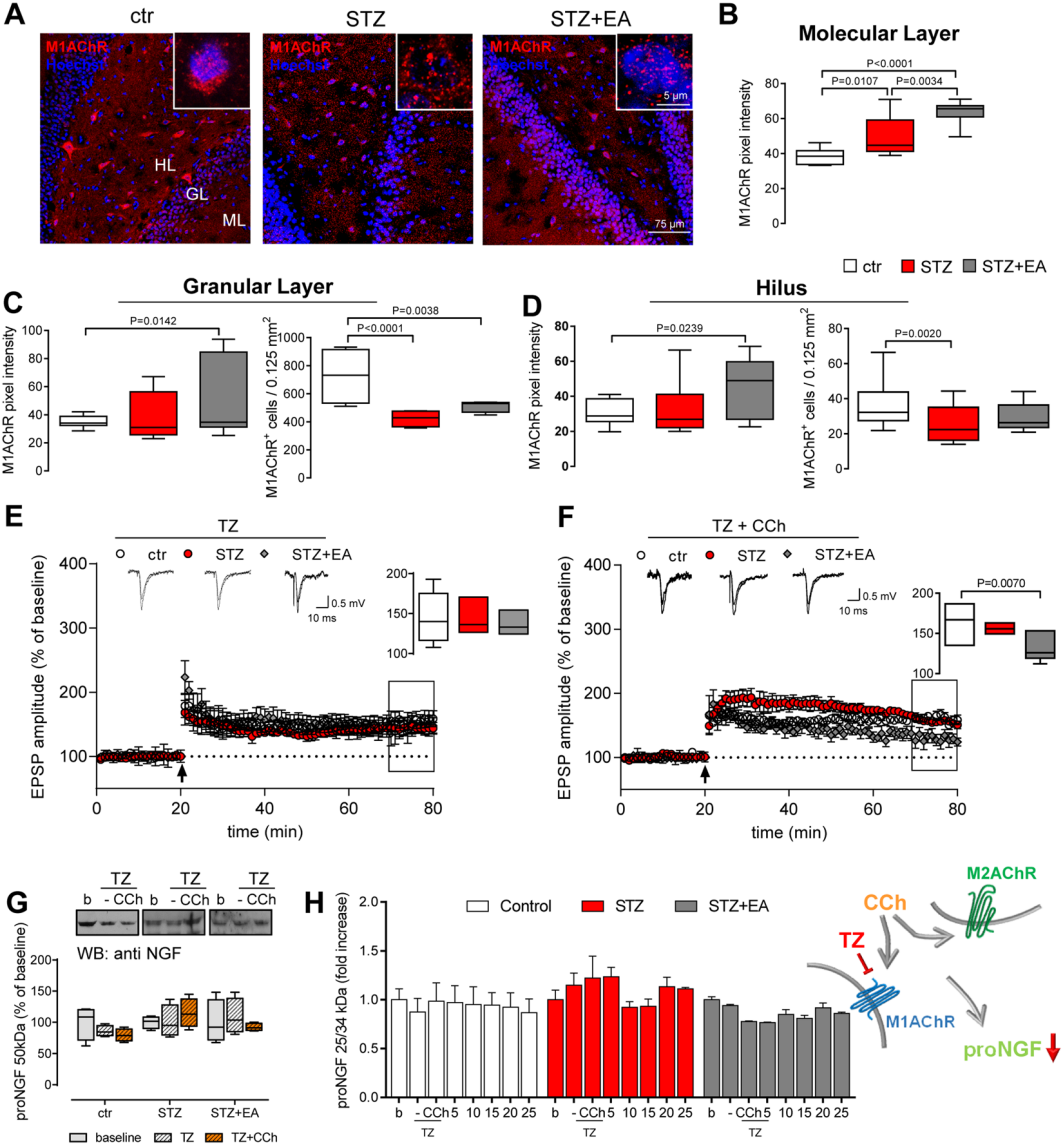
M1AChR tissue content and distribution is modified by diabetes and electroacupuncture and influences LTP and proNGF secretion. (**A**) Representative images of M1AChR immunolocalization in the dentate gyrus (DG). Insets: High-magnification details of M1AChR cellular distribution. (**B–D**) Mean pixel intensity and number of M1AChR immunopositive (M1AChR^+^) cells in different areas of the DG. Means ± SEM (n = 8 fields, 4 animals for each experimental group). One-way ANOVA followed by Bonferroni multiple comparison test, P values shown in figure. (**E,F**) Long-term potentiation at dentate gyrus (DG-LTP) after stimulation of hippocampal slices with the M1AChR selective antagonist telenzepine (TZ) and the non-specific muscarinic agonist carbachol (CCh). Field excitatory postsynaptic potentials (fEPSP) were recorded and expressed as the percentage of the pre-tetanus baseline. Changes in fEPSP slopes before and 60 min after the induction of LTP by high-frequency stimulation (HFS) of the medial perforant pathway (black arrow) are shown (means ± SEM; n = 8 for each experimental group). The insets represent typical fEPSP recordings; calibration bars: 0.5 mV, 10 ms. The average of DG-LTP magnitude 50–60 min after HFS is presented on the right side of the panels (median ± interquartile range, whiskers: min. and max.). One-way ANOVA and Bonferroni multiple comparison (P values shown in figure). (**G**) Carbachol-stimulated proNGF release from hippocampal slices. Telenzepine was added to the superfusion bath before hippocampal slices were exposed to carbachol. Densitometry of the Western blots (cropped representatives shown in the upper side of the panel; full-length blots are shown in online Supplementary material) illustrates the differences in the telenzepine/carbachol-stimulated proNGF release among the three experimental groups. Data are expressed as percentage of the respective baselines (median ± interquartile range, whiskers: min. and max.; n = 6 for each experimental group). Repeated-measure ANOVA followed by Bonferroni multiple comparison; P values shown in figure. (**H**) Carbachol-stimulated 25/34 kDa proNGF release from hippocampal slices measured by proNGF ELISA. Telenzepine was added to the superfusion bath before hippocampal slices were exposed to carbachol. Data, representing the percentage of the respective baseline means, are expressed as means ± SEM (n = 6 for each experimental group). Repeated-measure ANOVA followed by Bonferroni multiple comparison; P values shown in figure. Abbreviations: ML-molecular layer, GL-granular layer, HL-hilus, b-baseline, w/o-washout.

Since proNGF release is under cholinergic control, we explored the role of the two main muscarinic acetylcholine (ACh) receptors expressed in the hippocampus, M1AChR and M2AChR, after diabetes induction and/or electroacupuncture. In Fig. 4A, representative images show hippocampal sections immunolabeled for M1AChR. The faint punctate immunolabeling sparse among the cells, observed in the ctr group, increased in the STZ and STZ+EA groups. Differently, the intracellular immunolabeling in both HL and GL neurons decreased after diabetes induction and slightly recovered after electroacupuncture. An increased immunolabeling, as mean pixel intensity, was evident after streptozotocin, which however reached significance only in the ML (Fig. 4B–D). Electroacupuncture significantly enhanced the M1AChR immunostaining intensity in the ML (Fig. 4B–D). The number of M1AChR^+^ cells, in the GL and HL, decreased in the STZ group (Fig. 4C,D) and did not recover in GL after electroacupuncture (Fig. 4C). Thus, after diabetes induction, M1AChR increased and underwent tissue re-distribution, with a prevalent re-location of immunoreactivity toward the ML, while electroacupuncture further increased M1AChR protein in the DG.

We, then, verified whether M1AChR participates in the regulation of both DG-LTP and proNGF release by hippocampal cells. To this aim, we used telenzepine (TZ), a selective M1AChR antagonist, both in basal conditions and in the presence of carbachol. Bath-applied telenzepine (200 nM) lowered the magnitude of DG-LTP (Fig. 4E; statistics in Table 1), in hippocampal slices, from healthy rats (Table 1, row 3 *vs* row 1). Telenzepine did not affect DG-LTP in the STZ group, while it abolished the rescue of LTP (see Fig. 2F) in the STZ+EA group (Table 1 row 3 *vs* row 1), suggesting that M1AChR participates in the electroacupuncture-induced recovery of diabetes-impaired LTP.

Telenzepine counteracted the effects of carbachol on DG-LTP in healthy rats (Table 1, row 4 *vs* row 2), further confirming the LTP facilitation played by M1AChR. In the STZ group, carbachol together with telenzepine did not affect the magnitude of DG-LTP (Table 1 row 4 *vs* row 2). DG-LTP was depressed by carbachol and telenzepine, in the STZ+EA group (Fig. 4F; Table 1, row 4), suggesting a recovery of M1AChR activity after electroacupuncture. Furthermore, telenzepine exacerbated the carbachol-induced decrease in the magnitude of DG-LTP observed in the STZ+EA group (Table 1, row 4 *vs* row 2), indicating that electroacupuncture probably acts also on the muscarinic LTP-depressive component^27^.

Telenzepine did not affect the carbachol-stimulated release of the 50 kDa proNGF from hippocampal slices (Fig. 4G), in all of the experimental groups. The 25/34 kDa proNGF species in the superfusion media, measured by proNGF ELISA (Fig. 4H), were also not significantly modulated by carbachol after telenzepine. Of note, telenzepine pre-treatment inhibited the carbachol-induced increase in proNGF release (depicted at Fig. 3I), suggesting a main role for M1AChR in regulating the proNGF secretion from brain cells. A significant interaction effect was observed for the two variables (antagonist/time and experimental group) in the two-way ANOVA (Supplementary Table 2), suggesting that the activity of M1AChR was different in different experimental groups. Indeed, the presence of telenzepine in the superfusion bath, further inhibited the carbachol-induced proNGF release from STZ+EA compared to STZ hippocampi, as confirmed by Bonferroni multiple comparison between the STZ and STZ+EA groups (not shown in Fig. 4H).

### Role of M2AChR in modulating DG-LTP and proNGF release after diabetes and electroacupuncture

**Figure 5:**
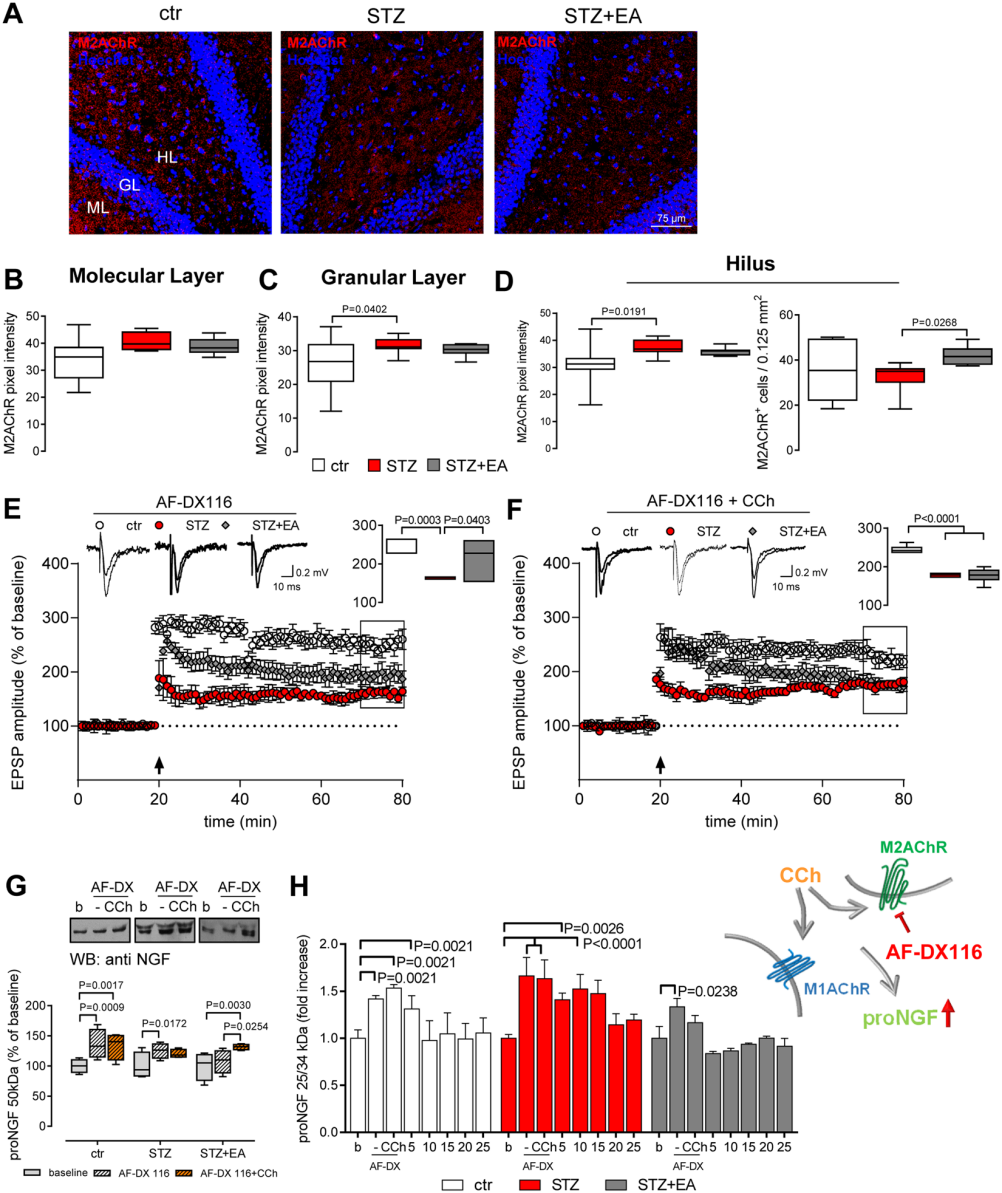
Diabetes- and electroacupuncture-modified M2AChR expression and its influence on LTP and proNGF secretion. (**A**) Representative images of M2AChR immunolocalization in the dentate gyrus (DG) of the hippocampus. (**B–D**) Mean pixel intensity of M2AChR immunostaining in different areas of the DG and number of M2AChR immunopositive (M2AChR^+^) cells in the hilus. No immune-positive cells were detected in the granular layer (GL). Means ± SEM (n = 8 fields, 4 animals for each experimental group). One-way ANOVA followed by Bonferroni multiple comparison test, P values shown in figure. (**E**,**F**) Long-term potentiation at DG (DG-LTP) after stimulation of hippocampal slices with the M2AChR selective antagonist AF-DX116 and the nonselective muscarinic agonist carbachol (CCh). Field excitatory postsynaptic potentials (fEPSP) were recorded and expressed as the percentage of the pre-tetanus baseline. Changes in fEPSP slopes before and 60 min after the induction of LTP by high-frequency stimulation (HFS) of the medial perforant pathway (black arrow) are shown (means ± SEM; n = 8 for each experimental group). The insets represent typical fEPSP recordings; calibration bars: 0.5 mV, 10 ms. The average of DG-LTP magnitude 50–60 min after HFS is presented on the right side of the panels (median ± interquartile range, whiskers: min. and max.). One-way ANOVA and Bonferroni multiple comparison, P values shown in figure. (**G**) Carbachol-stimulated proNGF release from hippocampal slices. AF-DX116 was added to the superfusion bath before hippocampal slices were exposed to carbachol. Densitometry of the Western blots (cropped representatives shown in the upper side of the panel; full-length blots are shown in online Supplementary material) illustrates the differences in AF-DX116/carbachol-stimulated proNGF release among experimental groups. Data are the percentage of respective baselines (median ± interquartile range, whiskers: min. and max.; n = 6 for each experimental group). Repeated-measure ANOVA followed by Bonferroni multiple comparison, P values shown in figure. **(H)** Carbachol-stimulated 25/34 kDa proNGF release from hippocampal slices measured by proNGF ELISA. AF-DX116 was added to the superfusion bath before hippocampal slices were exposed to carbachol. Data are percentage of the respective baseline and are expressed as means ± SEM (n = 6 for each experimental group). Repeated-measure ANOVA followed by Bonferroni multiple comparison, P values shown in figure. Abbreviations: ML-molecular layer, GL-granular layer, HL-hilus, b-baseline, w/o-washout.

We also evaluated the distribution and intensity of immunolabeling of the G_i/0_-coupled M2AChR in the hippocampus. M2AChR-immunopositive fibres decorated the extracellular parenchyma in both ML and HL, living the GL largely unlabelled. After diabetes induction, the intensity of immunolabeling increased (Fig. 5A), as confirmed by the mean pixel intensity in the GL and HL (Fig. 5C,D). Electroacupuncture treatment, in diabetic rats, did not affect the intensity of immunolabeling but increased the number of M2AChR^+^ cells in the HL (Fig. 5D). This suggests that electroacupuncture was promoting a re-distribution of the M2AChR after it was increased by diabetes in the DG.

We evaluated the possible functional correlates of the M2AChR re-distribution by measuring basal and carbachol-stimulated DG-LTP and proNGF release in the presence of the M2AChR selective antagonist 11-[[2-[(Diethylamino)methyl]-1-piperidinyl]acetyl]-5,11-dihydro-6H-pyrido[2,3-b][1,4]benzodiazepin-6-one (AF-DX116)^27^. AF-DX116 potentiates basal DG-LTP in healthy rats (Table 1, row 5 *vs* row 1). In the presence of AF-DX116, DG-LTP was depressed in slices from the STZ group (Fig. 5E; Table 1, row 5) and recovered to control level in those from the STZ+EA group (Fig. 5E; Table 1, row 5). These data indicate that the M2AChR-mediated DG-LTP modulation is normally depressive and non-significantly affected by diabetes and electroacupuncture.

The simultaneous presence of AF-DX116 and carbachol (Fig. 5F) induced a little, but significant, potentiation of DG-LTP in diabetic animals (Table 1, row 6 *vs* row 5), that was also noticed after carbachol administration alone (Table 1, row 6 *vs* row 2). In the STZ+EA group, the application of AF-DX116+carbachol depressed DG-LTP to STZ group levels (Fig. 5F; Table 1, row 6). This result confirms the apparent paradox effect of carbachol when applied to hippocampal slices from electroacupuncture-treated rats (Table 1, row 2 *vs* row 1). It suggests that the depression of LTP could be mediated by receptors other that M2AChR when an excess of agonist is present, which could be either endogenous acetylcholine or exogenous carbachol^27^. Taken together with the M1AChR data, a picture emerges where electroacupuncture recovered the overall muscarinic facilitatory response.

We, then, evaluated the release of proNGF in the presence of AF-DX116. The basal release of the 50 kDa proNGF, from hippocampal slices, after AF-DX116 (Fig. 5G) increased in both ctr and STZ groups, and was not affected in the STZ+EA group, suggesting that the acetylcholine present in the brain parenchyma tonically inhibits proNGF release by challenging M2AChRs. The exposure of brain slices to AF-DX116+carbachol increased the 50 kDa proNGF release in ctr and STZ+EA groups (Fig. 5G). AF-DX116 (before carbachol application) increased the 25/34 kDa proNGF in the superfusates in all of the experimental groups (Fig. 5H), further indicating that an M2AChR-mediated mechanism is active in controlling basal proNGF release, being probably not affected by both diabetes and electroacupuncture. Subsequent exposure of brain slices to carbachol, in the presence of AF-DX116, induced a transient proNGF release, which rapidly returned to baseline in ctr and STZ+EA groups, but remained higher than baseline for a longer time in the STZ group (Fig. 5H). The transient increase in proNGF release, in STZ group, was longer than the one shown in Fig. 3I, suggesting that the ability of M1AChR to stimulate proNGF secretion, selected by M2AChR blockade and carbachol challenge, was enhanced in diabetic brain and rescued by electroacupuncture.

## Discussion

**Figure 6:**
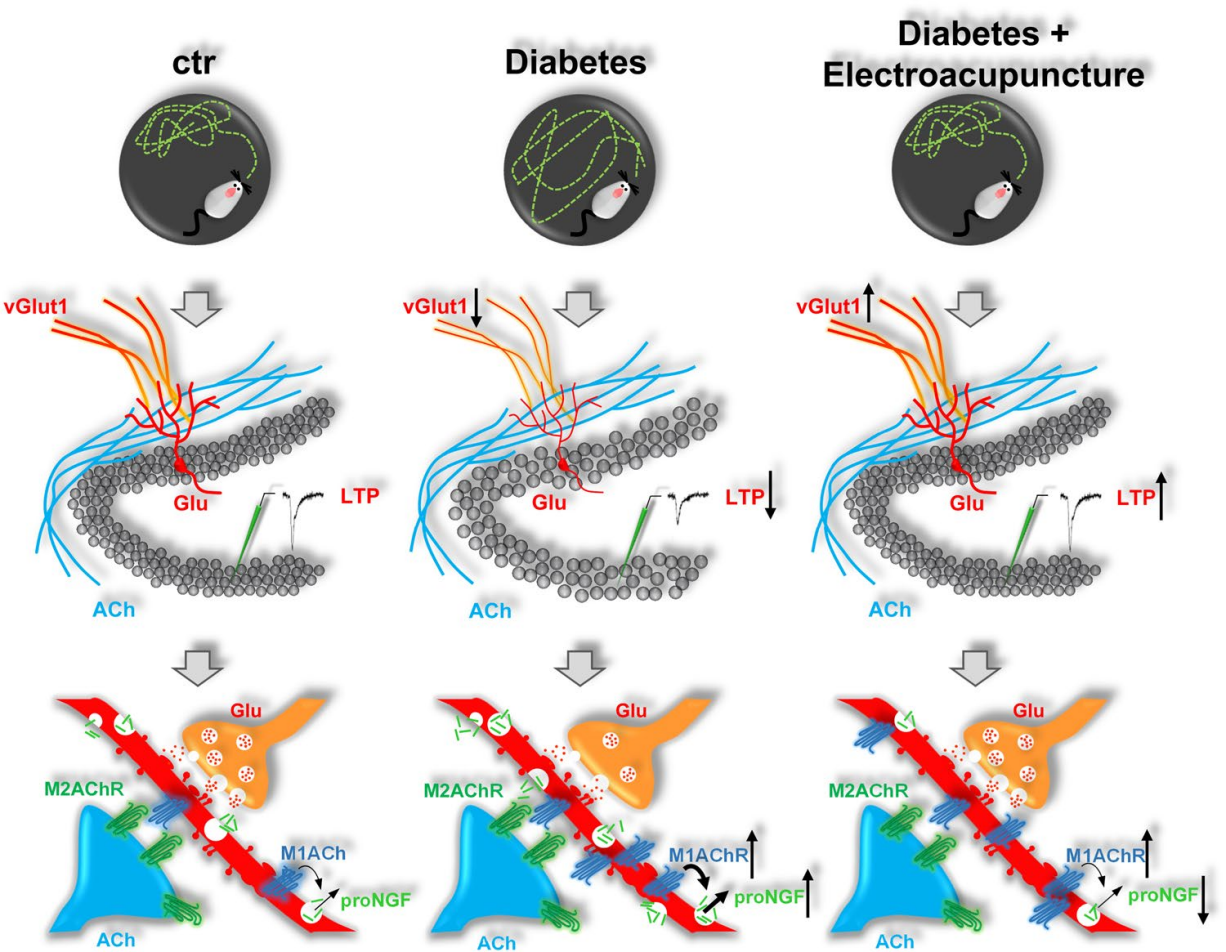
Summary drawing of the experimental findings. The behavioural alteration found in the Morris water maze early after diabetes induction in young adult rats (upper part of the figure), were concomitant to the reduction in glutamatergic transmission (middle part of the figure), neuronal loss in the hippocampus, the depression of synaptic plasticity and the increased expression and possibly activity of the M1-subtype muscarinic receptor, resulting in increased activity dependent release of the proNGF (lower part of the figure). The electroacupuncture normalized spatial learning and memory performance in diabetic rats, improved glutamatergic neurotransmission, LTP and the activity-dependent release of proNGF, modulating the response of muscarinic receptor to acetylcholine challenge.

It is relevant that a major therapeutic approach to delay and/or attenuate the cognitive decline, in both Alzheimer’s disease and diabetic encephalopathy, relies on physical therapies^28, 29^. Here, we demonstrate that the diabetes development in young adult rats is characterized by mild-to-severe neuronal loss in the hippocampus; impairment in hippocampal-related learning and memory; reduction in cellular glutamate trafficking and in synaptic plasticity; dysfunctions in the tissue distribution of muscarinic receptors; increased activity-dependent release of proNGF. Electroacupuncture, a physical therapy based on repetitive low-frequency sensory stimulation^16^, was able to counteract the early depressive effects of diabetes on the hippocampal neurotransmission and metabolism. Electroacupuncture, probably by rescuing the glutamate vesicular transport content and M1AChR-mediated cholinergic neurotransmission, normalized learning and memory functions and DG-LTP and regulated proNGF release in diabetic hippocampus. A schematic summary of the overall experimental findings is presented at Fig. 6.

The hippocampus is particularly vulnerable to both hyper- and hypoglycaemia^2, 30^. Our data on nuclear size and cell number indicate a possible general increase in transcription activity, confirming the apoptotic process activation in diabetic hippocampus^31^. The diabetes-induced cell loss encompassed both neuronal and non-neuronal cells, but the electroacupuncture rescuing effects appeared restricted to areas with high neuronal density (GL and PCL). Since electroacupuncture is a modulator of brain activity^32^ and neural plasticity^33, 34^, it is conceivable that excitatory granular and pyramidal neurons could benefit from the activation of sensory processes, that stimulates the recovery of diabetes-impaired excitatory transmission and the normalization of the muscarinic responsivity to cholinergic inputs. The stimulation delivered by electroacupuncture is known to modulate the peripheral and central processing of sensory information^16, 32^. The hippocampus integrates sensory information driven from cortical areas and the midbrain and brainstem, these latter conveyed through BFCNs^35, 36^. Thus, electroacupuncture could affect hippocampal functions, by modulating both subcortical and cortical hippocampal inputs.

The effects of diabetes and electroacupuncture were evident at the behavioural level. Diabetes induced a significant decline in spatial learning and memory functions and, following electroacupuncture, the abilities of spatial localization and memory reverted to control values. In the current experimental model of early diabetes, cognitive (spatial) deficits are present, similar to the dysfunctions in specific cognitive domains described in diabetic children and adolescents^37^. We demonstrate that they could benefit from electroacupuncture, which acts on specific functional and biochemical alterations in hippocampal regions. Our findings extend previous reports describing that nine weeks of hyperglycaemic pre-diabetic state are sufficient to generate mild cognitive impairment^38^, while 40-day streptozotocin treatment combined to a cerebral ischemia produces consistent learning and memory deficits^20^. Such a cognitive impairment is positively affected by electroacupuncture treatment^20^.

We characterized the diabetes-induced re-distribution of major players involved in activity-dependent hippocampal functions: vGlut1, M1AChR and M2AChR. vGlut1, a glutamate transporter preferentially associated with the membranes of synaptic vesicles and a specific molecular marker of vesicular glutamate release^39^. It has been suggested that vGlut1 has a functional role in hippocampal synaptic plasticity and in spatial learning and memory^40^. We found that vGlut1 was decreased in diabetic brain at sites where glutamatergic synapses participate in the propagation of excitatory signals from the cortex toward the hippocampus, namely ML, HL and PCL. Consistently, lower extracellular glutamate content at the DG of streptozotocin-treated rats has been previously reported^41^. The increase in the intracellular vGlut1 (at least in the HL) suggests that diabetes could selectively interfere with the traffic of glutamate toward efferent terminals, with a consequent decreased capability of diabetic hippocampus to generate LTP. Streptozotocin administration affected M1AChR and M2AChR in an opposite fashion in comparison to vGlut1. The suffering hippocampal neurons could undergo functional modifications^24^ and structural derangement, i.e. dendritic tree alteration^42^. In diabetic rats, these modifications may result in M1AChR protein delocalization from neuron somata in the DG to their dendrites in the ML. Here is where the transmission of excitatory signal from the cortex to the DG physically occurs at axo-dendritic contacts and where the cholinergic fibres from the BFCN also exert their modulatory activity^43^. M2AChR immunolabeling also increased, but in different areas than M1AChR, namely the GL and HL, coherently with M2AChR main role as regulator of interneuron activities^44^. Thus, in the early diabetes, there could be an attempt of hippocampal cells to compensate the impairment of glutamatergic transmission by increasing cholinergic sensitivity. This attempt, instead of ameliorating the excitatory function, could in turn generate a potential harmful effect, mediated by the proNGF released upon muscarinic challenge^10, 45^.

Our data put together the impairment in LTP, the excess of proNGF and the muscarinic responsivity generated by diabetes in the hippocampus. The secretion of proNGF is regulated by glutamatergic and/or cholinergic neurotransmission^9, 45^. It is conceivable that a M1AChR-mediated mechanism in the diabetic hippocampus is mostly responsible for the increase in proNGF tissue content^6^. Indeed, the reduction in glutamatergic transmission in the DG of diabetic rats was concomitant to an increased release of proNGF after muscarinic receptors challenge, an effect normalized by the M1AChR antagonist telenzepine. Considerably, the extracellular proNGF maturation and mNGF degradation^10^ was possibly shifted toward the latter (increased MMP9 activity in diabetic rats) that toward proNGF maturation (decrease of plasminogen and tPA in diabetic rats). proNGF accumulation, in diabetic hippocampus, could be involved in the regulation of neural plasticity and learning, directly influencing hippocampal cells and circuitries and/or by modulating cholinergic transmission from the BFCN^11, 46^. While mature NGF positively affects LTP generation through TrkA^46^, the role of proNGF in regulating DG-LTP remains elusive. A mechanism based on its action on p75^NTR^ deserves future in-depth analysis, in view of recent data demonstrating that proBDNF negatively regulates dendritic complexity and spines density and impairs hippocampal LTP by challenging p75^NTR^ ^47^, and that proBDNF-p75^NTR^ pathway is responsible for LTD-generated hippocampal synapse elimination^48^.

We hypothesized that electroacupuncture modulates hippocampal function trough the muscarinic activity. Both M1AChR and M2AChR positively regulate DG-LTP^25, 27^. M2AChR, acting as presynaptic auto-receptor, could also block the acetylcholine-mediated potentiation of LTP, by inhibiting acetylcholine release from cholinergic terminals^49^. The non-selective muscarinic agonist carbachol generated a large enhancement in DG-LTP in control but not in diabetic rats. Moreover, carbachol abolished the effects of electroacupuncture on diabetic hippocampi, leading the DG-LTP at levels comparable to those of the STZ group. This apparent paradox could be explained dissecting the respective roles of the muscarinic receptors. Telenzepine acted in similar ways in controls and STZ+EA groups while it was almost ineffective on hippocampi from STZ group, suggesting that electroacupuncture restores M1AChR modulation of DG-LTP. Conversely, blockade of M2AChR was ineffective in both STZ and STZ+EA groups. M2AChR located on interneurons inhibits GABA release, while on pre-synaptic cholinergic terminals it acts as inhibitory auto-receptor^49^. In our experiments, GABA-A receptors were blocked by picrotoxin to allow the measure of DG-LTP. Thus, the activity of AF-DX116 was restricted to the pre-synaptic M2AChRs and probably resulted in a facilitation of acetylcholine release by cholinergic terminals upon stimulation of the perforant pathway. This response was lacking in diabetic animals, independently of electroacupuncture treatments, suggesting that electroacupuncture did not influence M2AChRs activity. It is conceivable, however, that electroacupuncture restores the acetylcholine tissue content, since the concomitant stimulation of the perforant pathway and the exposure to carbachol led to muscarinic agonist high dosage-dependent depression in fEPSP^27^. These described effects should be most probably mediated by receptors other than M2AChR and not related to the GABA ergic transmission^27^.

We found that M1AChR stimulated proNGF release, while M2AChR inhibited it. The abnormal release of proNGF in diabetic hippocampi, as well as its increased tissue content^6^, could reflect increased M1AChR and/or reduced M2AChR activities. Electroacupuncture most probably normalizes this unbalance. Indeed, telenzepine depressed while AF-DX116 enhanced the carbachol-induced proNGF release, mainly in the STZ group; the STZ+EA group was instead found very similar to control. A muscarinic-mediated mechanism could be also responsible for the unbalance in the activity of the extracellular protease machinery responsible for proNGF maturation and mNGF degradation^10^, which shifted toward the latter in diabetic animals, probably contributing to the decrease in mNGF/proNGF balance in the diabetic brain^6^.

In conclusion, our data indicate that electroacupuncture in early diabetic rats, acts by favouring the maintenance of synaptic plasticity and its functional correlate (DG-LTP) in the hippocampus (Fig. 6). Electroacupuncture modulates both protein expression and hippocampal distribution of muscarinic receptors and of the neurotrophin proNGF, both involved in the regulation of hippocampal functions. It is conceivable that electroacupuncture action extends to the entire septo-hippocampal circuitry, also influencing the metabolism and functional properties of BFCNs. Overall, our findings point to the validity of physical therapies, such as electroacupuncture, as simple and effective interventions supportive of more complex and sometimes invasive pharmacological approaches targeting central neurons for the care of neurodegenerative diseases.

## Methods

Detailed experimental procedures are described in the Supplementary material.

### Animals

Fifty-day old female Sprague–Dawley rats were purchased from Harlan (Nossan, Italy). Rats were weighed and housed three per cage, with standard food and water available ad libitum. The animal room had a controlled 12-hours light cycle (lights on at 07:00 h), lux level (on average 100 lux), temperature (21 ± 1 °C) and relative humidity (50 ± 5%). All experiments were conducted according to the ARRIVE guidelines^50^. Animal care procedures were conducted in conformity with the Legislation for the protection of animals used for scientific purposes provided by the relevant Italian law and European Union Directive (Italian Legislative Decree 26/2014 and 2010/63/EU) and the International Guiding Principles for Biomedical Research involving animals (Council for the International Organizations of Medical Sciences, Geneva, CH)^51^. Animals were subjected to experimental protocols approved by the Veterinary Department of the Italian Ministry of Health (Permit Number: 192/2015–PR). All adequate measures were taken to minimize animal pain or discomfort and all surgery was performed under isoflurane anesthesia.

### Diabetes induction and experimental design

Sixty-day old female Sprague-Dawley rats received a single intraperitoneal injection of 65 mg/kg Streptozotocin (STZ; cat. S0130, Sigma-Aldrich), dissolved in citrate buffer (vehicle), pH 4.5^22^. One week after streptozotocin treatment, we analysed blood glucose and allocated rats with levels above 300 mg/dl to the diabetic groups. Rats were divided in three groups as follows: controls (ctr), once injected with vehicle; diabetics (STZ), treated with streptozotocin; electroacupuncture-treated diabetics (STZ+EA): electroacupuncture treatment was started one week after streptozotocin and performed twice a week for three weeks. Four weeks after streptozotocin, rats were either subjected to behavioural tests or euthanized and the whole brain or brain tissues collected for storage or immediate analysis.

### Electroacupuncture

Stainless steel needles (diameter 0.20 mm) were inserted bilaterally at the traditional Chinese acupoints “Stomach 36” and “Large Intestine 4”^5^. Low-frequency electroacupuncture was given to conscious rats, placed in a soft fabric harness and suspended above the desk^52^, through a specific electrical stimulator (CEFAR ACU II; Cefar-Compex Scandinavia). The acupoints were electrically stimulated at 2 Hz frequency with 0.1-sec, 80-Hz burst pulses. The intensity (0.8–1.0 mA) was monitored by checking for local muscle contractions, which reflect the activation of muscle-nerve afferents. Control and diabetic rats were exposed to the same handling and suspension procedure, but not to electroacupuncture.

### Behavioural testing in the Morris water maze

Seven rats/group were tested in the MWM apparatus, following a 3-day protocol. On day 1, each rat was submitted to 10-trial (120 s) Place 1 phase with the hidden platform put in the NW quadrant, followed by one trial (60 s) with no platform in the pool (Short-Term Probe 1, Probe ST1). On day 2, the rat was submitted to 10-trial Place 2 phase with the hidden platform put in the SE quadrant, followed by one Probe trial (Short-Term Probe 2, Probe ST2). On day 3, the rat was submitted to one Probe trial (Long-Term Probe, Probe LT). MWM parameters considered were: latencies to reach the platform (Place 1 and Place 2); distance swum in the previously rewarded (platform) quadrant (Probe ST1, Probe ST2 and Probe LT).

### Electrophysiology

For extracellular recordings, hippocampal slices (350 μM thick) were kept submerged at 30 °C and superfused (2–3 ml/min) with oxygenated (95% O_2_, 5% CO_2_) artificial CSF. Stimulation was applied to the medial perforant pathway of the dentate gyrus (DG), using a bipolar insulated tungsten wire electrode, and field excitatory postsynaptic potentials were recorded at a control test frequency of 0.033 Hz from the middle one-third of the molecular layer of the DG with a glass microelectrode. LTP was evoked by high-frequency stimulation (HFS) consisting of eight trains, each of eight stimuli at 200 Hz, and an inter-train interval of two seconds, with the stimulation voltage increased during the HFS protocol. Measurements of LTP were made 60 min post-HFS. All solutions contained 50 μM picrotoxin (P1675, Sigma-Aldrich) to block GABA-A-mediated activity. The muscarinic modulation of LTP was investigated by carbachol (100 nM; cat. C4382, Sigma-Aldrich) stimulation of hippocampus slices. The specific role of muscarinic receptors subtypes was studied by telenzepine (200 nM; cat. T122, Sigma-Aldrich) and AF-DX116 (200 nM; cat. SML0435, Sigma-Aldrich), respectively M1AChR and M2AChR selective antagonists, applied in bath superfusion with or without carbachol.

### proNGF release experiments

Hippocampal slices (350 µm thickness) were kept for 1h in cold oxygenated artificial CSF. Slices were then placed into a superfusion system (Minucells and Minutissue), using 5.0 µm Durapore™ membrane filters (cat. SVLP01300, Millipore). The tissues were constantly superfused, at 37 °C and a flow rate of 0.25 ml/min, with modified Hank’s buffer pH 7.8, equilibrated with 95% O_2_ and 5% CO_2._ After 30 min, a sample of the superfusion buffer was collected representing the baseline and a 5 minutes-long stimulation with 100 nM carbachol and/or with 200 nM telenzepine and/or with 200 nM AF-DX116 was applied, before returning to superfusion with modified Hank’s buffer. Samples were collected at 5 min intervals for 1h and immediately frozen at −80 °C.

### Immunofluorescence, Stereology and Confocal Microscopy

Coronal 40 μm-thick brain sections were pre-incubated with PBS containing 10% (v/v) donkey serum, 1% (w/v) BSA and 0.3% (v/v) Triton X-100, for 2 h at room temperature (RT). Sections were then incubated, overnight (ON) at 4 °C, with primary antibodies diluted in the same medium (details at Supplementary Table 1). To assess for staining specificity, some of the sections were incubated in purified non-specific rabbit or mouse IgG. After washing with PBS, sections were incubated (2 h, RT) with specific secondary antibodies. Successively, sections were rinsed in PBS, incubated for 10 min with Hoechst for nuclei staining.

For stereological analysis, cell counting was conducted using an Axioskop 2 (Zeiss) fluorescence microscope interfaced with the Stereo Investigator software package (MicroBrightField, v11). ROI (Supplementary Fig. 1A) were outlined using a 4x objective lens and cell counting was performed using the Optical Fractionator probe at a higher magnification (100x oil-immersion objective lens) according to the setup reported in Supplementary Fig. 1B.

For confocal microscopy analysis, sections were viewed at a confocal laser scanning microscope (SP5, Leica Microsystems) under sequential mode, to avoid crosstalk between channels. Confocal image acquisitions were conducted so that all samples were imaged using consistent settings for laser power and detector gain. Boundaries and subdivisions of the brain structures were identified with reference to the Paxinos’ Rat Brain atlas^53^. Image analysis was performed by the Imaris Suite 7.4 software (Bitplane A.G.). To evaluate the mean pixel intensity and the number of cells in the different areas, a mask for each channel was manually drawn using the Imaris Surface module.

### Immunoprecipitation

Superfusates (0.6 ml) were pre-cleared for 1h with protein A/G Sepharose (cat. 20421, Thermo Scientific) and then immune-precipitated, ON at 4 °C, with 2 µg of NGF H20 antibody (Supplementary Table 1) bound to 50 µl of protein A/G Sepharose beads. After incubation, protein A/G-bound immune-complexes were washed three times with PBS, then suspended in 50 µl of 2X reducing sample loading buffer and boiled at 95–100 °C for 5 min to denature the protein and separate it from the Ab-protein A/G Sepharose bead complex. Sample were then processed for Western blot.

### Western blot

Twenty milligrams protein samples were resolved by 8–12% SDS-PAGE as described^5^. Proteins were transferred onto nitrocellulose membrane by ON blotting at 30 V, then rinsed in T-PBS (PBS + 1% Tween 20), blocked in T-PBS containing 5% non-fat dry milk and incubated, ON at 4 °C, with the primary antibodies (Supplementary Table 1). The blotted membranes were then washed in T-PBS, incubated with horseradish peroxidase-labelled secondary antibody (Supplementary Table 1) and developed with the enhanced chemiluminescence (ECL) detection system (WBKLS0500, Millipore). Gel densitometry was performed on scanned immunoblot images, using the ImageJ gel analysis tool. Full-lenght blots and loading controls corresponding to the cropped blots shown at Figs 3, 4 and 5 are depicted in Supplementary Fig. S4.

### proNGF ELISA

The proNGF content in superfusates was measured by a recently developed specific ELISA^6^. The capture and detection antibodies (Supplementary Table 1) were chosen to detect selectively proNGF and to avoid cross-detection of mature NGF.

### Statistical Analysis

Statistical analysis was performed using GraphPad Prism 5 (GraphPad Software). Means were generally compared by one-way ANOVA and, unless mentioned otherwise (i.e. in Table 1), multiple comparisons performed by Bonferroni post-hoc test. Differences were considered statistically significant if P < 0.05. When the measures for the main variable (three experimental groups) were repeated over time or after a pharmacologic treatment, means were analysed by two-way ANOVA with significance level = 0.05 (two-way ANOVA data at online Supplementary Table 2). Multiple comparisons by Bonferroni post-hoc test were then performed according to the main or interaction effects revealed by two-way ANOVA. Further details on Study design and statistics are given in online Supplementary methods.

## Electronic supplementary material

Supplementary Information
