# Supplementary material for: Recovery of hippocampal functions and modulation of muscarinic response by electroacupuncture in young diabetic rats
Source: Sci Rep. 2017 Aug 22;7:9077. doi: 10.1038/s41598-017-08556-z (PMC5567336; doi:10.1038/s41598-017-08556-z)
Supplement: Supplementary file 1 — Supplementary Information [file 41598_2017_8556_MOESM1_ESM.pdf]

Recovery of hippocampal functions and modulation of muscarinic response by electroacupuncture in young diabetic rats

Marzia Soligo[1], Sonia Piccinin[2], Virginia Protto[1], Francesca Gelfo[3,4], Maria Egle De Stefano[5], Fulvio Florenzano[2], Erica Berretta[3,6], Laura Petrosini[3,6], Robert Nisticò[7], Luigi Manni[1,*]

[1] *Institute of Translational Pharmacology, Consiglio Nazionale Delle Ricerche (CNR), Rome, Italy*

[2] *European Brain Research Institute (EBRI), Rita Levi-Montalcini Foundation, Rome, Italy*

[3] *I.R.C.C.S., Santa Lucia Foundation, Rome, Italy*

[4] *Department of Systemic Medicine, University of Rome "Tor Vergata", Rome, Italy*

[5] *Department of Biology and Biotechnology "Charles Darwin", Sapienza University of Rome, Laboratory affiliated to Istituto Pasteur-Fondazione Cenci Bolognetti, Italy*

[6] *Department of Psychology, Faculty of Medicine and Psychology, University "Sapienza" of Rome, Rome, Italy*

[7] *Department of Biology, University of Rome Tor Vergata, Rome, Italy*

SUPPLEMENTARY INFORMATION

Supplementary Methods

Study Design. This study explored the efficacy of a physical therapy, the electroacupuncture, in counteracting the deleterious effects of early diabetes on hippocampal physiology. We also investigated the hypothesis that the muscarinic activity in the hippocampus mediated the therapeutic effects of electroacupuncture. All experiments were conducted according to the ARRIVE guidelines[1]. Randomization was carried out using the online Research Randomizer Program (www.randomizer.org), so that the persons carrying out behavioral or electrophysiology analysis, tissue processing and statistical analysis were blind to the treatment. The GPower program (http://www.gpower.hhu.de/en.html) was used to calculate the sample size, according to the statistical methodologies used to compare means in the different experiments (see Statistical analysis section below). Differences among the compared means ≥ 30% with SD ≤ 20% of the mean within groups have been considered to obtain a power of at least 80% with an alpha level of 0.05.

Animals. Sixty-day old female Sprague–Dawley rats were purchased from Harlan (Nossan, Correzana, Italy). Rats were weighed, ear tagged and housed in groups of three per cage with standard food and water available ad libitum. The animal room had a controlled 12-hours light cycle (lights on at 07:00h), lux level (on average 100 lux), temperature (21 ± 1°C) and relative humidity (50 ± 5%). Animal care procedures were conducted in conformity with the Legislation for the protection of animals used for scientific purposes provided by the relevant Italian law and European Union Directive (Italian Legislative Decree 26/2014 and 2010/63/EU) and the International Guiding Principles for Biomedical Research involving animals (Council for the International Organizations of Medical Sciences, Geneva, CH)[2]. Animals were subjected to experimental protocols approved by the Veterinary Department of the Italian Ministry of Health (Permit Number: 192/2015–PR). All adequate measures were taken to minimize animal pain or discomfort and all surgery was performed under isoflurane anesthesia.

Diabetes induction and experimental design. Streptozotocin was used to produce an animal model of type 1 diabetes[3]. Sixty-day old rats, from different litters, received a single intraperitoneal injection of 65 mg/kg streptozotocin (cat. S0130, Sigma-Aldrich, Milan, Italy), dissolved in citrate buffer, pH 4.5. One and four weeks after streptozotocin treatment, we checked the establishment of diabetes with an Accutrend™ GC (Roche Diagnostic, Germany) glucose analyzer. Rats with blood glucose levels above 300 mg/dl were allocated to the diabetic experimental groups. Seventy-five rats were randomly divided in three groups as follow: 25 control rats

were injected once i.p. with 20 mmol/l citrate buffer, pH 4.5 (ctr group) and 50 rats were injected with streptozotocin as described above (STZ groups; n=25). Electroacupuncture in diabetic animals (STZ+EA group; n=25) was repeated twice a week for 3 weeks, starting one week after diabetes induction[4,5]. For memory function evaluation, seven rats for each experimental group were tested in the Morris Water Maze (MWM), 4 weeks after streptozotocin treatment. For electrophysiology experiments, 4 weeks after streptozotocin treatment, eight rats for each experimental group were euthanized by decapitation, after mild anesthesia by isoflurane. After decapitation, the brain was removed and 350 µm-thick parasagittal sections from both hemispheres were cut by vibratome. For superfusion experiments, 4 weeks after streptozotocin treatment, six rats for each experimental group were euthanized by decapitation, under light isoflurane anesthesia. The brains were rapidly dissected at 4°C and cut by vibratome. Four rats for each experimental group were used for immunofluorescence, stereology and confocal microscopy analysis. After surgical deep anesthesia, rats were intra-cardially perfused by 4 % paraformaldehyde and brains dissected and further processed as described below.

Electroacupuncture. From a Western perspective, electroacupuncture is a non-pharmacological method known to trigger a number of reactions at spinal level and in the brain[6,7]. Low-frequency electroacupuncture (1-4 Hz), with intensity high enough to cause muscle twitches, probably excites low and high threshold mechanoreceptors[8] and particularly a group of receptors found in muscles, indicated as ergoreceptors[9,10] that are physiologically activated during muscle contractions. It has been suggested that electroacupuncture, with repetitive muscle contraction, results in the activation of physiological processes similar to those resulting from physical exercise[6]. Electroacupuncture stimulation was achieved by inserting stainless steel needles (diameter 0.20 mm) bilaterally at the traditional Chinese acupoints Stomach 36 (ST36; at the proximal insertion of the tibialis anterior muscle) and Large Intestine 4 (LI4; in the middle of the right dorsal thenar muscle), known to be effective in the activation of central and peripheral NGF system[4,5]. From a Western perspective, it has been found that both of them are particularly effective when a central response to acupuncture treatment is needed, especially in modulating the limbic system activity[11]. Low-frequency electroacupuncture was given to conscious rats, placed in a soft fabric harness and suspended above the desk[12], through a specific electrical stimulator (CEFAR ACU II; Cefar-Compex Scandinavia, Malmo, Sweden). The acupoints were electrically stimulated at 2 Hz frequency with 0.1-sec, 80-Hz burst pulses. The intensity (0.8 – 1.0 mA) was monitored by checking for local muscle contractions to reflect the activation of muscle-nerve afferents (A-delta fibers and possibly C-fibers). The location and type of stimulation

were the same in all animals. Rats were stimulated by electroacupuncture twice per week for 3 weeks, starting one week after diabetes induction. Control and diabetic rats were exposed to the same handling and suspension procedure but not to electroacupuncture.

Based on our previous studies and supported by the current knowledge about the use of proper controls in acupuncture studies, we decided not to enroll, in our experimental setup, the control + electroacupuncture group as well as any kind of minimal or sham-electroacupuncture-treated diabetic rats[5,13-15]. Controls are key factor in acupuncture studies[14,15]. It is actually recognized that there is no ideal control treatment in acupuncture studies[15]. The evidence that needling activates all of the possible afferent fibers type, vanish the use of every control treatment based on minimal, superficial or sham stimulation. However, it has been suggested that most of the effects of acupuncture are stress-induced[15]. Thus, where applicable to experimental design, rodents subjected to manipulation procedure, but not to acupuncture, are used as treatment controls. Moreover, as for its effects on healthy subjects, the acupuncture results in increased activity in the limbic structures whereas in patients with pain a deactivation of the same structures is reported[15]. This would suggest that studies using healthy subjects is of great interest but have limited relevance when a disease-specific therapeutic action of electroacupuncture is investigated.

Behavioural testing in the Morris water maze (MWM). After the end of the electroacupuncture treatment, to test amnesic spatial functions, seven rats for each group were tested in the MWM. The rats were placed in a circular white pool (diameter 140 cm) located in a normally equipped laboratory room, uniformly lighted by four neon lamps (40 W each). Extra-maze spatial cues were on the walls and held in constant spatial relations throughout the experiments. The pool was filled with 24 ± 2°C water (60 cm deep), made opaque by the addition of atoxic acrylic black color (Giotto, Italy). An escape platform (diameter 10 cm) was placed in the middle of one quadrant (NW, SE), 30 cm from the side walls. It was either submerged 2 cm or raised 2 cm above the water level. Testing was performed between 09:00 AM and 05:00 PM hours.

The protocol kept three days. On day 1, each rat was submitted to 10-trial Place 1 phase (hidden platform put in the NW quadrant). The last trial was followed by 1 trial with no platform in the pool (Short-Term Probe 1 phase – Probe ST1). On day 2, each rat was submitted to 10-trial Place 2 phase (hidden platform put in the SE quadrant). The last trial was followed by 1 trial with no platform in the pool (Short-Term Probe 2 phase – Probe ST2). On day 3, each rat was submitted to 1 trial with no platform in the pool (Long-Term Probe phase – Probe LT).

The rat was released into the water from randomly varied starting points and allowed to search for the hidden or visible platform for a maximum of 120 s. When the rat reached the platform, it was allowed to remain there for 30 s. The inter-trial interval was 60 s. In the Probe phases the platform was removed and rats were allowed 60 s to search for it. The rats' trajectories in the pool were monitored by a video camera mounted on the ceiling. The resulting video signal was relayed to a monitor and to the image analyzer (Ethovision, Noldus, Wageningen, The Netherlands). As behavioral parameters in analyzing performances, latencies to reach the platform (Place 1 and Place 2 phases) and total distance swum in the previously rewarded (platform) quadrant (Probe ST1, Probe ST2 and Probe LT phases) were considered. As for the latencies, analyses were executed taking into account mean values obtained by grouping the trials in 5 sessions (2 trials/session).

Immunofluorescence, Stereology and Confocal Microscopy. Serial 40 µm-thick coronal sections of both hemispheres, that spanned -2.40/-3.72 mm relative to Bregma, according to Paxinos' Rat Brain atlas[16], were cut on a cryostat and then processed. In preparation for an unbiased stereological estimate of cell numbers, an initial tissue section was selected randomly at one anatomic border of the brain region to be estimated. Brain sections were pre-incubated with PBS containing 10 % (v/v) donkey serum, 1% (w/v) BSA and 0.3% (v/v) Triton X-100 for 2h at room temperature (RT). Sections were then incubated overnight (ON) at 4°C with primary antibodies diluted in the same medium (for details see Supplementary Table 1). To assess for staining specificity, some of the sections were incubated in purified non-specific rabbit or mouse IgG. After washing with PBS, sections were incubated (2h, RT) with specific secondary antibodies. Successively, sections were rinsed three times in PBS and incubated for 10 min with Hoechst for nuclei staining.

Stereological quantification of Hoechst$^+$ and NeuN$^+$ cells was performed online using the Stereo Investigator software (v11, MBF Bioscience, VT, USA). The stereological analysis was carried out with 10 sampling intervals between sections using an Axioskop 2 (Carl Zeiss S.p.A., Italy) fluorescence microscope interfaced with the Stereo Investigator software package. ROI were outlined using a 4x objective lens (Supplementary Fig. 1A) and cell counting was performed using the Optical Fractionator probe at a higher magnification (100x oil-immersion objective lens). A 100 x 100 µm grid with a 20 x 20 µm counting frame was systematically and randomly superimposed on ROI. The Optical Disector height was 20 µm with a 5 µm guard zone. Cell counting was performed on three sections for each ROI, that is considered sufficient to provide a coefficient of error between

0.09 and 0.12. Only cells within the counting frame or overlapping the green right or superior border of the counting frame, and for which nuclei came into focus while focusing down the disector height, were counted. The measure used for calculating the Optical Fractionator results was the "estimated cell population using mean section thickness with counts". Parameters of stereological analysis are detailed in Supplementary Fig. 1B.

For confocal microscopy vGlut1, M1AChR, M2AChR, proNGF and doublecortin (DCX) distribution in the dentate gyrus of the hippocampus were analyzed. One section every 160 μm (1 in 4 sections) throughout the anatomic region of interest was processed for each staining series and viewed at a confocal laser scanning microscope (Leica SP5, Leica Microsystems, Germany) under sequential mode, to avoid crosstalk between channels. Confocal image acquisitions were conducted so that all samples were imaged using consistent settings for laser power and detector gain. Boundaries and subdivisions of the brain structures were identified with reference to the Paxinos' Rat Brain Atlas[16]. Image processing was done by using the Adobe Photoshop CS6 software: production, image brightness and contrast were enhanced by using the linear histogram correction and slightly oversaturated. Image analysis was performed by the Imaris Suite 7.4 software (Bitplane A.G., Switzerland) on eight different images derived from each group. For all image-processing steps, images were compared with the original raw data to make sure that no structures were introduced that were not seen in the original data series or that structures present in the original data series were not removed. To evaluate the mean pixel intensity and the number of cells in the different areas, four non-overlapping region of interest (ROI) were manually drawn using the Imaris Surface module. Imaris software was used to automatically count the number of labeled cells in each ROI after establishing a detection threshold, which was kept constant within each measurement.

Western blot. Samples were treated with 4X reducing sample loading buffer (62.5 mM Tris HCl pH 6.8, 20 % (v/v) glycerol, 8 % (w/v) SDS, 0.025% (w/v) bromophenol blue and 100 mM dithiothreitol) and boiled at 95-100°C for 5 min. Samples were resolved by 8-12 % SDS-PAGE – using a Bio Rad Mini Protean 3 Cells – at 25-30 mA in running buffer (25 mM Tris HCl pH 8.3, 190 mM Glycine, 0.1% SDS). Proteins were blotted onto nitrocellulose membrane ON at 30 V in blotting buffer (25 mM Tris HCl pH 8.3, 190 mM Glycine, 20% methanol). Blots were then rinsed in T-PBS (PBS + 1% Tween 20), blocked in T-PBS containing 5% non-fat dry milk 1h at RT and then incubated with the primary antibodies, indicated in Supplementary Table 1, ON at 4°C. The blotted membranes were then extensively washed in T-PBS at RT, incubated with horseradish peroxidase (HRP)-labeled secondary antibody (Supplementary Table 1) and developed with the enhanced chemiluminescence's (ECL) detection

system (cat. WBKLS0500, Millipore). Gel densitometry was performed on scanned immunoblot images using the ImageJ gel analysis tool (https://imagej.nih.gov/ij/). Full-lenght blots and loading controls corresponding to the cropped blots shown at Fig. 3, 4 and 5 are depicted in Supplementary Fig. S4.

proNGF ELISA. To measure the proNGF content in superfusates, we used a recently developed specific ELISA[17]. The capture antibody (Supplementary Table 1) was incubated ON at RT, into the wells of Nunc™ MaxiSorp™ ELISA Plates (cat. 439454, Thermo Fisher Scientific). Unbound antibody was removed by washing the plate once with washing buffer (0.5% (v/v) Triton X-100 in PBS). After blocking 1h at RT with PBS + 1% (w/v) BSA, the plate was rinsed with washing buffer and superfusates or standard curves added to the wells and incubated for 2h at RT. The microwells were then rinsed three times and incubated with detection antibodies dissolved in blocking buffer for 2 h at RT. Following three wash to remove unbound detection antibody, HRP-conjugated antibody, diluted in blocking buffer, was added and incubated for 1h at RT. To visualize antibody reactivity, the chromogenic substrate 3',3',5',5'-tetramethylbenzidine (TMB, cat. T8768, Sigma-Aldrich) was used and color development was stopped by adding 1N HCl. The colorimetric reaction was measured in absorbance mode at 450 nm by a Multiskan EX ELISA reader (Thermo Fisher Scientific Laboratory).

Gelatin Zymography. Gelatin zymography was performed to detect biological activity of MMP-2 and MMP-9, as described[18]. Samples were resolved on non-reducing SDS-polyacrylamide gel containing 0.5 mg/ml gelatin. After electrophoresis, the gel was incubated in zymogram renaturing buffer (2.5 % (v/v) Triton X-100 in double distilled water) with gentle agitation for 1h at RT, then washed for 30 min in double distilled water before incubation for 18h at 37°C in developing buffer (50 mM Tris-HCl pH 7.4, 0.2 mM NaCl, 5 mM $CaCl_2$). The gel was then stained with Coomassie Brilliant Blue R-250 (cat. 161-0436, Bio Rad) for 30 min. Areas of protease activity appeared as clear bands against a dark blue background after incubation of the gel with Coomassie destaining solution (cat. 161-0438, Bio Rad).

Statistical analyses. Statistical analyses were performed using GraphPad Prism 5 (GraphPad Software). When the effect of diabetes induction and electroacupuncture were assessed at the end of experimental procedures (i.e. stereological analysis, confocal microscopy, probe tests in behavioral study), means were compared by one-way

ANOVA and multiple comparisons performed by Bonferroni post-hoc test. When the measures for the main variables (three experimental groups) were repeated over time or after a pharmacologic treatment (i.e. with muscarinic agonist or antagonist), means were analyzed by two-way ANOVA with family-wise significance level = 0.05 (see Supplementary Table 2 for a summary of two-way ANOVA data presented in this manuscript). Multiple comparisons by Bonferroni post-hoc test were then performed according to the main or interaction effects revealed by two-way ANOVA. Unpaired t-test was performed to compare the effects of the pharmacological stimulation/blockade of muscarinic receptors in the same experimental group in electrophysiology study (see Table 1). Data are presented as mean ± standard error of the mean (SEM) or median ± interquartile range (IQR). All analyses were two-tailed and P-values < 0.05 were considered statistically significant. Reported P-values were adjusted for multiple comparisons.

References

1 Kilkenny, C., Browne, W. J., Cuthill, I. C., Emerson, M. & Altman, D. G. Improving bioscience research reporting: the ARRIVE guidelines for reporting animal research. *PLoS biology* **8**, e1000412 (2010).

2 International Guiding Principles for Biomedical Research Involving Animals issued by CIOMS. *The Veterinary quarterly* **8**, 350-352 (1986).

3 Wilson, G. L. & Leiter, E. H. Streptozotocin interactions with pancreatic beta cells and the induction of insulin-dependent diabetes. *Curr Top Microbiol Immunol* **156**, 27-54 (1990).

4 Pagani, L., Manni, L. & Aloe, L. Effects of electroacupuncture on retinal nerve growth factor and brain-derived neurotrophic factor expression in a rat model of retinitis pigmentosa. *Brain Res* **1092**, 198-206 (2006).

5 Rocco, M. L. *et al.* Brain Cholinergic Markers and Tau Phosphorylation are Altered in Experimental Type 1 Diabetes: Normalization by Electroacupuncture. *J Alzheimers Dis* **33**, 767-773 (2013).

6 Andersson, S. & Lundeberg, T. Acupuncture - from empiricism to science: functional background to acupuncture effects in pain and disease. *Med Hypotheses* **45**, 271-281 (1995).

7 Sato, A., Sato, Y. & Uchida, S. Reflex modulation of visceral functions by acupuncture-like stimulation in anesthetized rats. *International Congress Series* **1238**, 111-123 (2002).

8 Noguchi, E. *et al.* The effect of electro-acupuncture stimulation on the muscle blood flow of the hindlimb in anesthetized rats. *J Auton Nerv Syst* **75**, 78-86 (1999).

9 Kaufman, M. P., Longhurst, J. C., Rybicki, K. J., Wallach, J. H. & Mitchell, J. H. Effects of static muscular contraction on impulse activity of groups III and IV afferents in cats. *Journal of applied physiology: respiratory, environmental and exercise physiology* **55**, 105-112 (1983).

10 Kniffeki, K. D., Mense, S. & Schmidt, R. F. Muscle receptors with fine afferent fibers which may evoke circulatory reflexes. *Circ Res* **48**, I25-31 (1981).

11 Wu, M. T. *et al.* Central nervous pathway for acupuncture stimulation: localization of processing with functional MR imaging of the brain--preliminary experience. *Radiology* **212**, 133-141 (1999).

12 Johansson, J. *et al.* Intense Acupuncture Normalizes Insulin Sensitivity, Increases Muscle GLUT4 Content, and Improves Lipid Profile in a Rat Model of Polycystic Ovary Syndrome. *Am J Physiol Endocrinol Metab* **299**, E551–E559 (2010).

13 Manni, L., Aloe, L. & Fiore, M. Changes in cognition induced by social isolation in the mouse are restored by electro-acupuncture. *Physiol Behav* **98**, 537-542 (2009).

14 Lund, I. & Lundeberg, T. Are minimal, superficial or sham acupuncture procedures acceptable as inert placebo controls? *Acupunct Med* **24**, 13-15 (2006).

15 Lundeberg, T., Lund, I., Sing, A. & Naslund, J. Is Placebo Acupuncture What It is Intended to Be? *Evid Based Complement Alternat Med* (2011).

16 Paxinos, G. *The rat brain in stereotaxic coordinates*. (Academic Press, 1982).

17 Soligo, M. *et al.* The mature/pro nerve growth factor ratio is decreased in the brain of diabetic rats: Analysis by ELISA methods. *Brain Res* **1624**, 455-468 (2015).

18 Leber, T. M. & Balkwill, F. R. Zymography: a single-step staining method for quantitation of proteolytic activity on substrate gels. *Anal Biochem* **249**, 24-28 (1997).

Supplementary Table 1: Antibodies used in different experiments.

Primary antibody (catalog, manufacture)	*Application /Dilution*	*RRID*	*Secondary antibody (catalog, manufacture, dilution)*	*Application*
guinea-pig **anti-vGlut1** (AB5905, Millipore)	IF: 1:900	AB_2301751	**Donkey-anti guinea pig Alexa-Fluor 555** (IS20276, Immunological Science, 1:200) RRID: AB_2571518	IF for vGlut1 content in HP slices: Fig. 2H
rabbit **anti-M1AChR** (sc-9106, SantaCruz)	IF: 1:100	AB_2244882	**Donkey-anti rabbit Alexa-Fluor 555** (A-31572, Thermo Fisher, 1:200) RRID: AB_2536182	IF for M1AChR content in HP slices: Fig. 4A
rabbit **anti-M2AChR** (sc-9107, SantaCruz)	IF: 1:100	AB_2080064	**Donkey-anti rabbit Alexa-Fluor 555** (A-31572, Thermo Fisher, 1:200) RRID: AB_2536182	IF for M2AChR content in HP slices: Fig. 5A
mouse ***anti-NeuN*** (MAB377, Millipore)	IF: 1:200	AB_2298772	**Donkey-anti mouse Alexa-Fluor 488** (A-21202, Thermo Fisher, 1:200) RRID: AB_2535788	IF for NeuN content in HP slices: Fig. S1
goat ***anti-DCX*** (sc-8066, SantaCruz)	IF: 1:200	AB_2088494	**Donkey-anti goat Alexa-Fluor 647** (A-21447, Thermo Fisher, 1:200) RRID: AB_2535864	IF for DCX content in HP slices: Fig. S1
rabbit **anti-proNGF** (AB9040, Chemicon)	IF: 1:400	AB_262175	**Donkey-anti rabbit Alexa-Fluor 488** (A-21206, Thermo Fisher, 1:200) RRID: AB_2535792	IF for proNGF content in HP slices: Fig. 3A
rabbit **anti-NGF H20** (sc-548, SantaCruz)	WB: 1:500	AB_632011	**HRP-conjugated anti-rabbit** (7074, Cell Signaling, 1:4000) RRID: AB_2099233	WB for proNGF content in superfusates: Fig. 3F, 3G, 4G, 5G,
	IP: 2 µg			proNGF isoform analysis (see: rabbit **anti-NGF H20 + protein A-peroxidase)**
	WB: 1:500		**Protein A-Peroxidase** (P8651, Sigma-Aldrich; 0.2 µg/ml)	WB for IP proNGF in superfusates: Fig. 3H
mouse **anti-plasminogen** (sc-69793, SantaCruz)	WB: 1:500	AB_1127306	**HRP-conjugated anti-mouse** (7076, Cell Signaling; 1:5000) RRID: AB_330924	WB for plasminogen content in superfusates: Fig. S2A
rabbit **anti-tPA** (PA5-27908, Pierce)	WB: 1:1000	AB_2545384	**HRP-conjugated anti-rabbit** (7074, Cell Signaling, 1:4000) RRID: AB_2099233	WB for tPA content in superfusates: Fig. S2B
Goat **anti-proNGF** (AF-556-NA, R&D)	ELISA: 0.4 µg/ml	AB_2298544		Used as Capture antibody: Fig. 3I, 4H, 5H
rabbit **anti-proNGF EP1318Y** (68151, Abcam)	ELISA: 1:5000	AB_11156445	**HRP-conjugated anti-rabbit** (7074, Cell Signaling, 1:1000) RRID: AB_2099233	Used as Detection antibody: Fig. 3I, 4H, 5H

IF: immunofluorescence, IP: immunoprecipitation, WB: Western blot

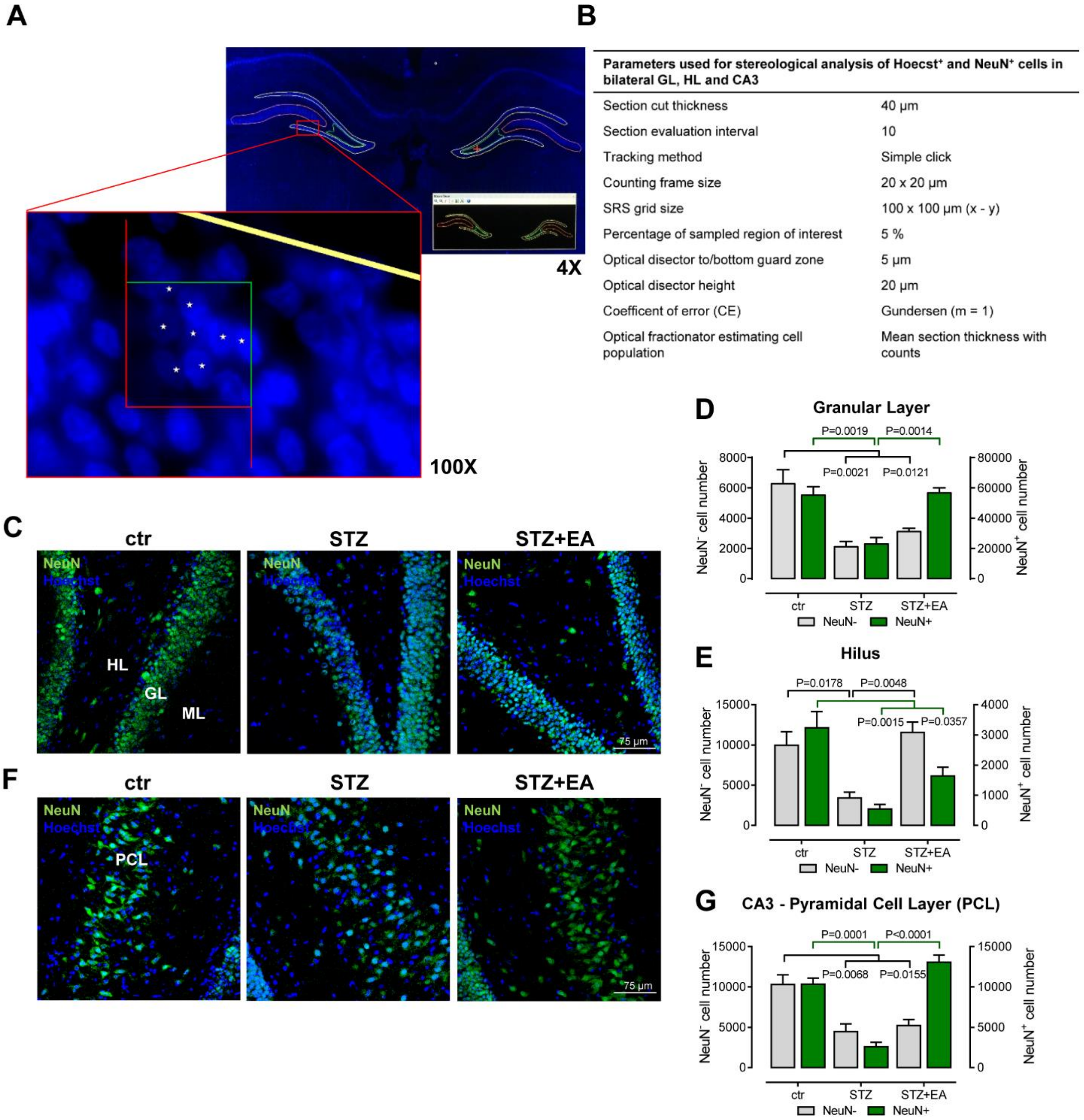

Parameters used for stereological analysis of Hoecst+ and NeuN+ cells in bilateral GL, HL and CA3	
Section cut thickness	40 µm
Section evaluation interval	10
Tracking method	Simple click
Counting frame size	20 x 20 µm
SRS grid size	100 x 100 µm (x - y)
Percentage of sampled region of interest	5 %
Optical disector to/bottom guard zone	5 µm
Optical disector height	20 µm
Coefficent of error (CE)	Gundersen (m = 1)
Optical fractionator estimating cell population	Mean section thickness with counts

Supplementary Fig. S1. Effects of diabetes and electroacupuncture on the number of neuronal and non-neuronal cells in the dentate gyrus. **(A)** Outlined DG areas used by the Stereo Investigator software to place counting frames (20 x 20 µm) within a randomly generated virtual grid (100 x 100 µm) for stereological quantification of Hoechst and NeuN$^+$ cells in the dentate gyrus volume spanned -2.40/-3.72 mm relative to Bregma (B) Parameters used for stereological analysis of Hoechst and NeuN$^+$ cells in bilateral granular layer (GL), hilus (HL) and CA3-pyramidal cell layer (PCL). **(C)** Representative images of NeuN immunolocalization in the dentate gyrus (DG) of the hippocampus. Sections were counterstained with the Hoechst nuclear dye to allow NeuN$^+$ and NeuN$^-$ phenotypes discrimination. **(D-E)** Quantification of NeuN$^+$ and NeuN$^-$ cells in the granular layer (GL) and hilus (HL) (means ± SEM of the estimated total cell number, n = 4 animals for each experimental group). One-way ANOVA followed by Bonferroni multiple comparison test, P values shown in figure. **(F)** Representative images of NeuN immunolocalization in the CA3 area of the hippocampus. Sections

were counterstained with the Hoechst nuclear dye so that $NeuN^+$ and $NeuN^-$ phenotypes can be discriminated. **(G)** Quantification of $NeuN^+$ and $NeuN^-$ cells in the pyramidal cell layer of the CA3 area (means ± SEM of the estimated total cell number, n = 4 animals for each experimental group). One-way ANOVA followed by Bonferroni multiple comparison test, P values shown in figure**.**

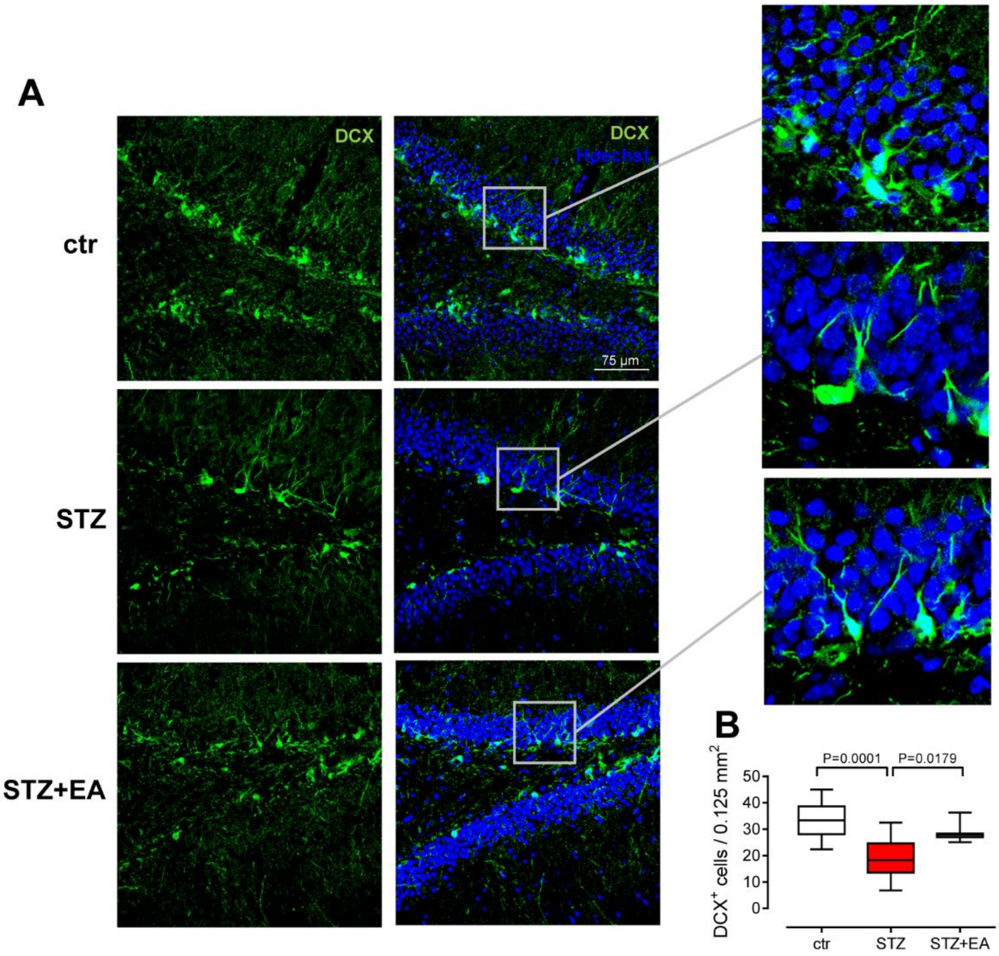

Supplementary Fig. S2. Effects of diabetes and electroacupuncture on the number of doublecortin cells in the dentate gyrus. **(A)** Representative images of doublecortin (DCX) immunolocalization in the sub-granular zone (SGZ) of the dentate gyrus (DG) of the hippocampus. Sections were counterstained with the Hoechst nuclear dye. **(B)** Quantification of DCX^+ cells in the SGZ (means ± SEM, n=8 fields, 4 animals for each experimental group). One-way ANOVA followed by Bonferroni multiple comparison test, P values shown in figure.

Supplementary Table 2: Summary of two-way ANOVA analyses.

Figure	*Time / muscarinic challenge*	*Diabetes and EA*	*Interaction*
1			
B	$F_{(1, 72)}$ = 0.7176; P = 0.3997	$F_{(2, 72)}$ = 234.0; P < 0.0001	$F_{(2, 72)}$ = 2.707; P = 0.0735
C	$F_{(2, 144)}$ = 1.421; P = 0.2449	$F_{(2, 72)}$ = 26.20; P <0.0001	$F_{(4, 144)}$ = 44.71; P < 0.001
2			
A	$F_{(4, 72)}$ = 0.4096; P = 0.8012	$F_{(2, 18)}$ = 6.260; P = 0.0086	$F_{(8, 72)}$ = 1.209; P = 0.3059
C	$F_{(4, 72)}$ = 3.945; P = 0.060	$F_{(2, 18)}$ = 4.795; P = 0.0214	$F_{(8, 72)}$ = 0.7590; P = 0.6396
3			
G	$F_{(1, 9)}$ = 59.04; P < 0.0001	$F_{(2, 9)}$ = 16.84; P = 0.0009	$F_{(2, 9)}$ = 8.350; P = 0.0089
I	$F_{(7, 105)}$ = 14.47; P < 0.0001	$F_{(2, 15)}$ = 1.001; P = 0.3906	$F_{(14, 105)}$ = 1.872; P= 0.0377
4			
G	$F_{(2, 18)}$ = 0.2111; P = 0.8116	$F_{(2, 9)}$ = 1.137; P = 0.3628	$F_{(4, 18)}$ = 1.468; P = 0.2533
H	$F_{(7, 105)}$ = 1.347; P = 0.2357	$F_{(2, 15)}$ = 1.464; P = 0.2626	$F_{(14, 105)}$ = 2.338; P = 0.0075
5			
G	$F_{(2, 18)}$ = 21.16; P < 0.0001	$F_{(2, 9)}$ = 0.5093; P = 0.6172	$F_{(4, 18)}$ = 2.629; P = 0.0687
H	$F_{(7, 105)}$ = 15.50; P < 0.0001	$F_{(2, 15)}$ = 3.641; P = 0.0514	$F_{(14, 105)}$ = 3.104; P = 0.0005
Suppl.2			
A	$F_{(1, 9)}$ = 14.97; P = 0.0038	$F_{(2, 9)}$ = 5.137; P = 0.0325	$F_{(2, 9)}$ = 15.37; P = 0.0013
B	$F_{(1, 9)}$ = 3.176; P = 0.1084	$F_{(2, 9)}$ = 2.330; P = 0.1530	$F_{(2, 9)}$ = 6.317; P = 0.0193

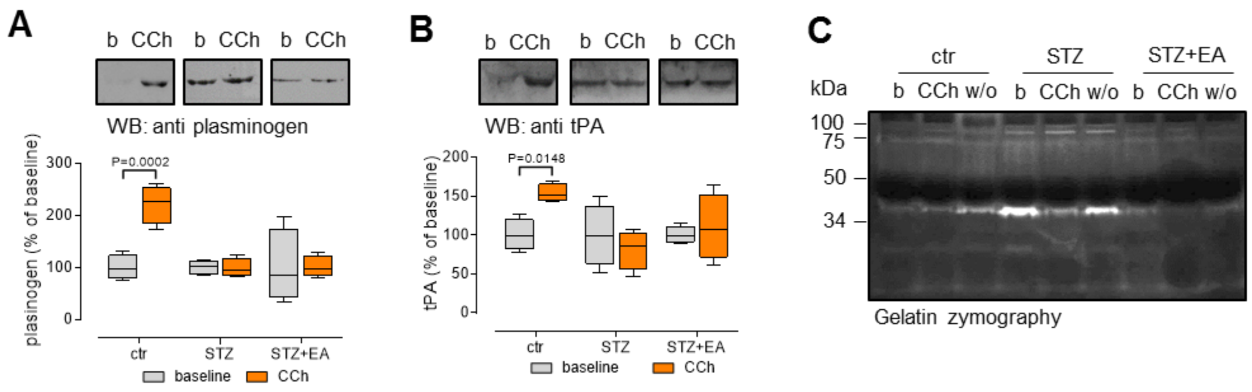

Supplementary Fig. S3. Diabetes and electroacupuncture treatment modify NGF levels by acting on the protease cascade responsible for proNGF maturation/degradation. (**A-B**) Representative Western blots (WB) in the upper side of the panel illustrates Carbachol (CCh)-stimulated plasminogen (**A**) and tPA (**B**) released from hippocampal slices. Densitometric analysis of four WBs is shown in the lower side of the panel. Data are presented as the % fold increase from baseline (median ± interquartile range, whiskers: min. and max.; n=4 for each experimental group). Two-way ANOVA followed by Bonferroni multiple comparison, P values shown in figure. (**B**) Activity of MMPs in the superfusion media revealed by gelatin zymography. MMP enzymatic activities increase in the hippocampus of streptozotocin treated rats (STZ group) compared to the baseline levels (b), while CCh stimulation induces a transient decrease in MMP activities in the superfusion media. Electroacupuncture treatment of diabetic rats (STZ+EA group) normalizes MMP activities in the superfusates toward control levels.

3F

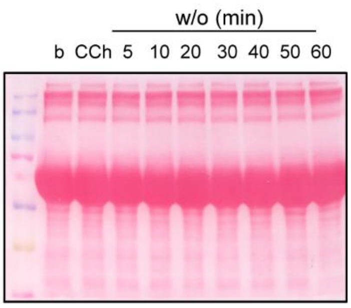
w/o (min)
b CCh 5 10 20 30 40 50 60

3H

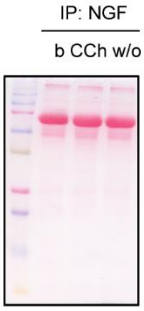
IP: NGF
b CCh w/o

3G

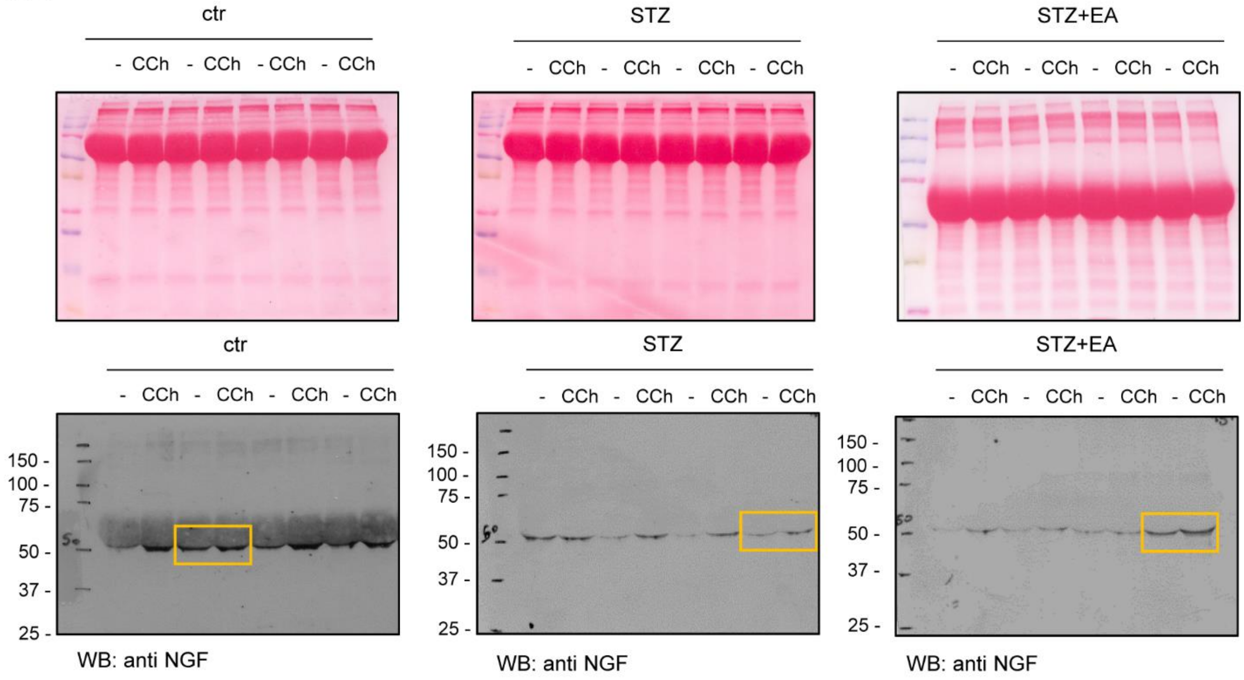
ctr
- CCh - CCh - CCh - CCh
STZ
- CCh - CCh - CCh - CCh
STZ+EA
- CCh - CCh - CCh - CCh
150 -
100 -
75 -
50 -
37 -
25 -
WB: anti NGF
WB: anti NGF
WB: anti NGF

4G

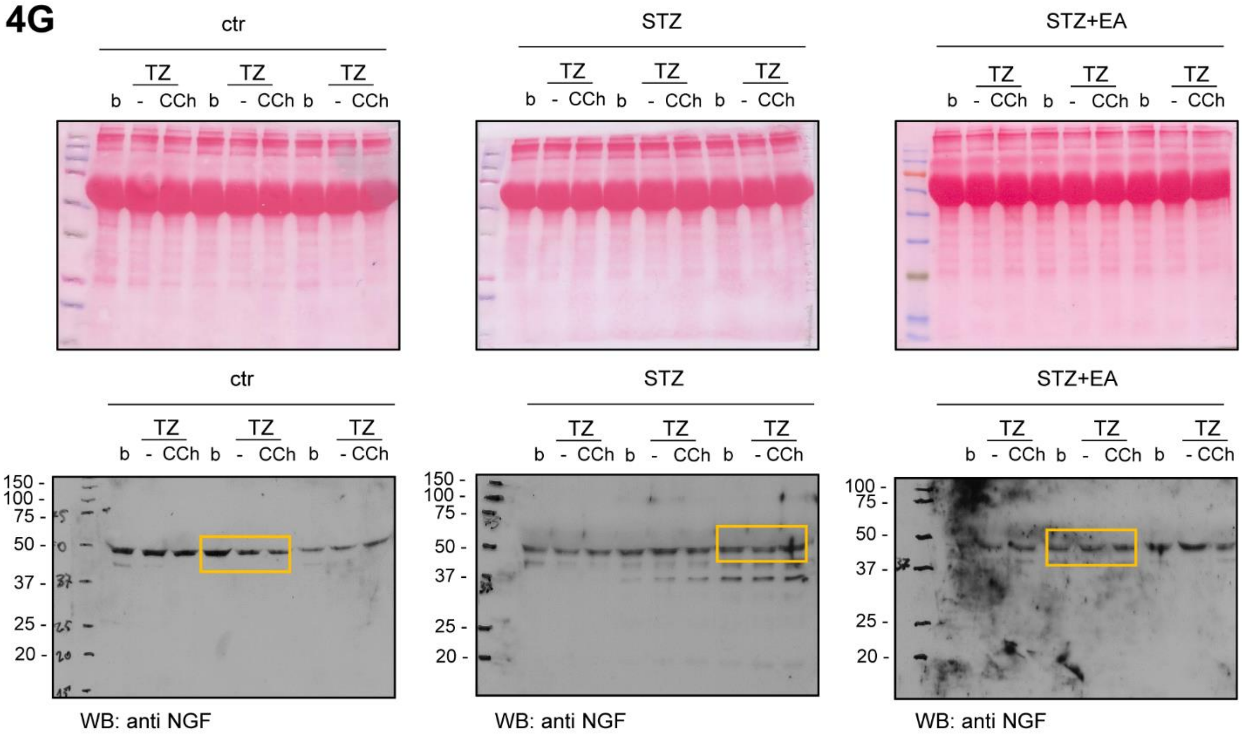
ctr
TZ TZ TZ
b - CCh b - CCh b - CCh
STZ
STZ+EA
150 -
100 -
75 -
50 -
37 -
25 -
20 -
WB: anti NGF
WB: anti NGF
WB: anti NGF

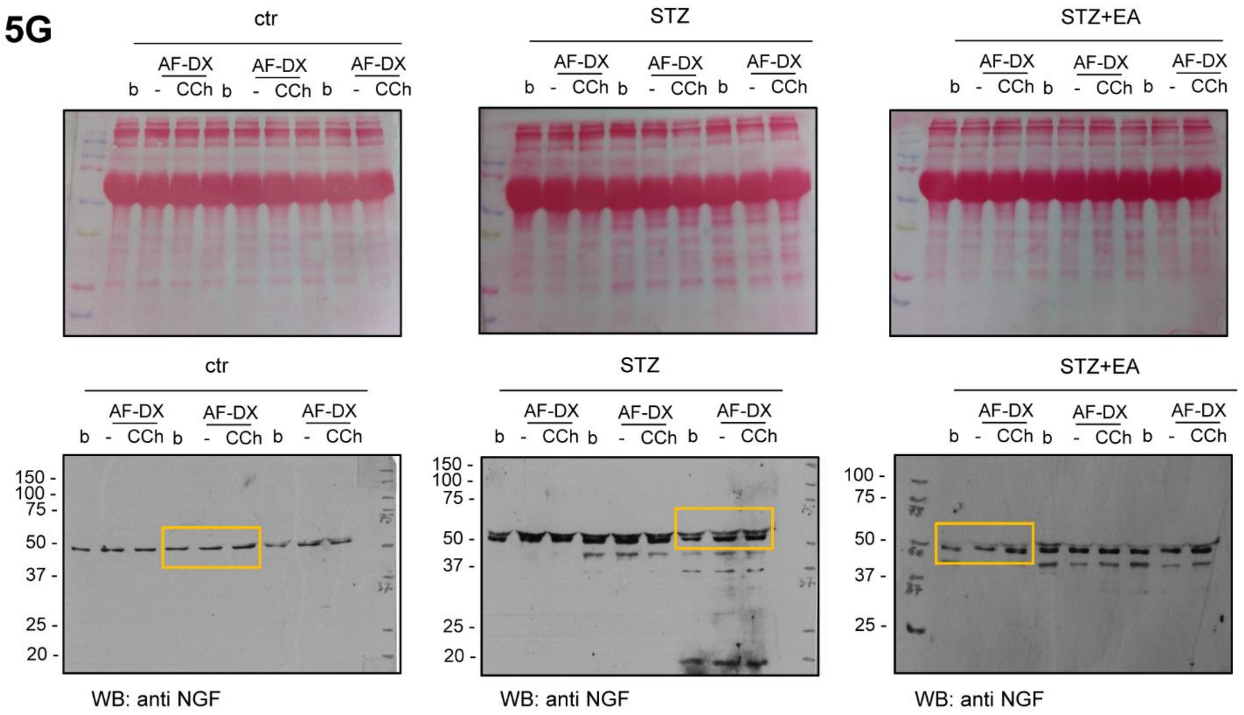

Supplementary Fig. S4. Full-lenght blots presented in Figures 3, 4, 5. Loading controls represented by Ponceau's staining of the membrane before immunoblot procedure and full length immunoblot relative to the main figure/panel indicated on the upper left side of each blot or blot series. The boxes evidence the representative lanes shown in the main figures. Abbreviations: b-baseline, CCh-carbachol, ctr-controls, STZ-streptozotocin-treated, STZ+EA-streptozotocin+electroacupuncture-treated, TZ-telenzepine, AF-DX-AF-DX116.
